# Flowing in the net of disordered gaming: A network analysis approach

**DOI:** 10.1016/j.abrep.2025.100606

**Published:** 2025-04-13

**Authors:** Tyrone L. Burleigh, Trent Footitt, Michelle Colder Carras, Connor Conkey-Morrison, Dylan R. Poulus, Vasileios Stavropoulos

**Affiliations:** a School of Health and Biomedical Sciences, RMIT University, Melbourne, Australia; b Nottingham Trent University, Nottingham, United Kingdom; c Johns Hopkins Bloomberg School of Public Health, Baltimore, United States; d Faculty of Health, Southern Cross University, East Lismore, Australia; e Movember Institute of Men’s Mental Health, Melbourne, Victoria, Australia

**Keywords:** Disordered gaming, Immersive gaming, Network analysis, Online flow, Time distortion

## Abstract

The experience of online flow, characterized by immersion and time distortion, may enhance gaming enjoyment but also contribute to disordered gaming patterns. The present paper examined online flow components and their associations with disordered gaming symptoms among 565 role-playing-gamers. Network analysis identified the centrality of time distortion and loss of control within flow states and highlighted their connections to disordered gaming indicators, such as impaired control and gaming prioritization. Enjoyment and positive challenge were core features within flow, suggesting they support adaptive engagement. However, pathways linking prolonged immersion with gaming disorder symptoms indicate that intense flow experiences may increase problematic gaming risks. These findings reveal a nuanced interaction between online flow and disordered gaming, where adaptive enjoyment and challenge can coexist with maladaptive control loss. Overall, the findings highlight how elements of flow can differentially affect gaming outcomes, contributing both to healthy engagement and potential disorder.

## Highlights

• Central elements in gaming flow are enjoyment and positive challenge.
• Time distortion is the strongest component in online flow experiences.
• Online flow and disordered gaming symptoms are distinct but interrelated.
• Weak ties exist between disordered gaming and flow’s enjoyment component.
• Implications suggest fostering flow elements to mitigate gaming disorder risks.

## 1 Introduction

In the last two decades, video games have become a popular positive and adaptive leisure activity engaged in by billions of people worldwide (Colombo et al., 2023, Stevens et al., 2023). However, for a minority of gamers, problematic or excessive gaming can lead to an array of negative mental and physical health outcomes, such as sleep disturbances, depression, anxiety, and stress (Billieux et al., 2019, Poulus et al., 2024, Stavropoulos et al., 2023, Wong et al., 2020). Consequently, problematic gaming has been provisionally defined within the Diagnostic and Statistical Manual of Mental Disorders (DSM-5-TR; American Psychiatric Association, 2022) as Internet Gaming Disorder (IGD), highlighting a need for additional clinical research. Whilst the global prevalence of IGD is estimated at around 1.96 %−3.05 % (Hu et al., 2021, Stevens et al., 2023), estimates vary greatly among different population and demographic groups (Wong et al., 2020, Zhang et al., 2022). As the percentage of gamers continues to increase year after year, new and engaging features within video games are continually developed to enhance player’s in-game experience, such as achievements, avatar personalization, and social collaboration can increase feelings of connection to and immersion within in-game worlds (Kiraly et al., 2023, Stavropoulos et al., 2022, Stavropoulos et al., 2023).

Collaboration in online social games, particularly prevalent in Massively Multiplayer Online games (MMOs), can be essential for achieving in-game objectives and has been demonstrated to enhance feelings of in-game immersion (Hu et al., 2019). Research by Hu et al. (2019; 2021) highlights that online social collaboration can lead to feelings of “immersive pleasure”, fostering beneficial social relationships and positively affecting affective states. When this immersive pleasure occurs alongside all-encompassing focus, and the challenge of the task meets the player’s skill level, a psychological state known as flow emerges (Hu et al., 2019, Hu et al., 2021). This optimally balanced state leads to effortless concentration and heightened satisfaction, improving feelings of experience and enjoyment (Csikszentmihalyi, 1990). Whilst primarily framed in literature as a positive state, the immersive experience of flow appears to be paradoxical in the context of gaming; sometimes referred to as “dark flow”, flow may not always necessarily be associated with positive outcomes (Csikszentmihalyi, 1990, Dixon et al., 2018, Larche and Dixon, 2021).

### 1.1 The paradox of flow in gaming

Flow is achieved when a gamer’s skill proficiency and task challenge are optimally balanced. An activity perceived as too easy may induce boredom, whilst an excessively difficulty task may evoke anxiety and disengagement, with the ideal state occurring when the challenge modestly surpasses the individual's skill level (Csikszentmihalyi, 1990). Further, concentration and immersion play pivotal roles in the state of flow, together implicating an intense focus that absorbs one's attention to the extent that the player loses awareness of external factors (Csikszentmihalyi, 1990, Bocci et al., 2023, Kiatsakared and Chen, 2022). The potential benefits of flow, such as heightened engagement and intense focus, can inform positive gaming experiences that enhance player well-being and cognitive processes (Hu et al., 2019, Jennett et al., 2008, Zairi et al., 2022). This is particularly relevant for emerging serious games (i.e., games designed primarily for educational, training, or informative purposes rather than entertainment), wherein flow can be strategically incorporated to enhance engagement, learning, and well-being, and foster knowledge retention and skill development (Buzady et al., 2022, Zairi et al., 2022). The heightened concentration required to achieve flow can improve the efficacy of educational content delivery and, in therapeutic contexts, can aid in creating engaging interventions for various health conditions (Zairi et al., 2022). Within social games (e.g., MMOs), flow has been associated with enjoyable, immersive experiences, enabling deep social connections that develop with increased time spent gaming (Antons et al., 2023, Hu et al., 2019, Kiatsakared and Chen, 2022, Li et al., 2021, Zhang et al., 2022).

Conversely, dark flow is characterized primarily by escapism coping behaviors and disruptions in affective balance (Dixon et al., 2018, Hu et al., 2021, Larche and Dixon, 2021). As with positive flow, immersion during dark flow is heightened, supported by a player’s bond with their avatar (Stavropoulos et al., 2023, Zhang et al., 2022), which can lead to time distortion and the prioritization of in-game tasks over real-life needs (Hu et al., 2021, Jennett et al., 2008, Kiatsakared and Chen, 2022). Indeed, Csikszentmihalyi (1990) posits that flow possesses inherently addictive qualities, and numerous studies have established a correlation between flow and increased gaming activity – yet conceptual understanding of flow remains ongoing (Hu et al., 2019, Li et al., 2021, Zhang et al., 2022). Further, distinctions between high usage and pathological usage are inconsistently defined, with high usage not necessarily indicative of negative usage (Billieux et al., 2019, Hu et al., 2021). Despite this, a potential causative relationship is implicated wherein the intrinsically rewarding nature of flow may contribute to excessive and problematic gaming behaviors (Hu et al., 2019, Kiatsakared and Chen, 2022, Li et al., 2021) Thus, the complex interplay of factors underlying flow and problematic gaming require nuanced exploration.

Individuals suffering from IGD have been shown to withdraw from real-life activities, seeking to satisfy psychological needs by engaging in compensatory behaviors within the in-game environment (Antons et al., 2023, Blasi et al., 2019, Li et al., 2021, Stavropoulos et al., 2023, Zhang et al., 2022). This prioritization of in-game activities over those in gamers’ real lives may result from heightened flow experiences during gaming sessions (Hu et al., 2021). Consequently, persistently seeking a flow state through engaging in high levels of gaming may become a maladaptive coping mechanism, especially if gaming tolerance increases without achieving the benefits of an optimal flow state (Hu et al., 2019, Hu et al., 2021, Li et al., 2021). This increased tolerance and preoccupation with gaming may lead to disruptions in affective balance and alterations in emotional regulation processes over time (Hu et al., 2019, Li et al., 2021). Nonetheless, most gamers do not experience problematic gaming behavior, and although increased usage remains a potential risk factor for IGD, flow is generally a positive experience (Hu et al., 2019, Larche and Dixon, 2021). As such, flow’s paradoxical nature within gaming underscores the intricate interplay between affective states, immersive experience, and potential adverse consequences associated with problematic gaming behaviors.

### 1.2 Defining flow in gaming

Interestingly, immersion has been described as a precursor to flow, though research that discriminates between each factor explicitly is inconsistent, particularly in gaming experiences (Colombo et al., 2023, Jennett et al., 2008). Indeed, extreme levels of immersion have also been described as flow, further complicating conceptual definitions and understanding (Kannegieser et al., 2019). These conceptual inconsistencies surrounding flow’s positive and negative effects necessitate deeper research to refine definitions and measures explicitly within the gaming context. Existing research has developed various scales and methodologies to assess flow, ranging from considering only a few key behavioral components to more comprehensive approaches encompassing a broader range of behaviors. For example, the Flow State Scale (FSS; Csikszentmihalyi & Csikszentmihalyi, 1988) and the Game Flow Scale (GFS; Sweester & Wyeth, 2005) both measure concentration, absorption, skill balance, clear goals, immediate feedback, intrinsic motivation, and positive affect. The FSS additionally measures time distortion and loss of self-consciousness. Variations in measurement approaches undermine the comparability of findings across studies and the generalizability of flow assessments to different and specific gaming contexts (Kannegieser et al., 2019). Moreover, distinguishing between the underlying factors of flow that contribute to both adaptive and maladaptive gaming experiences is essential for informing interventions targeting disordered gaming behaviors.

While correlations between flow and disordered gaming are well-established (Hu et al., 2019; Hu et al., Li et al., 2021; 2021; Zhang et al., 2022), understanding the specific components of flow that drive adaptive gaming experiences versus those that contribute to maladaptive patterns remains a critical research gap. Exploring how flow manifests in online gaming compared to offline contexts is crucial for advancing understanding of flow’s nuanced dynamics. The unique features of online gaming, such as real-time social interactions and immersive virtual environments, may influence the experience of flow in distinct ways that require careful consideration.

### 1.3 Present study

Thus, by implementing a network analysis methodology, the present research aims to identify the components that constitute online flow and determine whether it functions as a formative and/or reflective construct. By examining the central and peripheral features inherent in the experience of online flow and investigating potential overlaps with disordered gaming behaviors using a network analysis approach, the study provides a robust examination of reflective and formative constructs by analysing interconnections among various components. This will help extrapolate the central features driving flow experiences. This investigation is crucial for advancing the understanding of the nuanced dynamics between Flow experiences and potential disordered gaming patterns.

## 2 Methods

The present study is part of a larger international project examining longitudinal risk factors for gaming disorder. The current paper specifically analyzes gaming disorder and online flow, focusing on cross-sectional associations between individual items of these constructs. This publicly accessible dataset (see Stavropoulos et al., 2023) has already contributed to a number of prior publications (e.g., Brown et al., 2024, and Hein et al., 2024).

### 2.1 Participants

A 6-month longitudinal study investigated gaming patterns and demographic profiles in an online community. Data collection took place at two separate time points, gathering responses from 627 participants. Following exclusions—preview-only responses (n = 7), spam (n = 19), bots (n = 1), cases without consent (n = 12), failed validity responses (n = 8), and insufficient responses (n = 15)—the final dataset included 565 adolescents and adult role-playing-gamers (Mage = 29.3 years, SD = 10.6; 50 % male, 45 % female, 4 % other gender; 16 % adolescents [n = 89], 84 % adults [n = 473]). The present paper utilizes the first wave of data for these participants, with initial demographics and gaming behaviors summarized in Table 1, Table 2.

**Table 1:** Participant’s age, game playing/social media usage years and daily week and weekend time.

		Age		Gaming Years		Mean Daily Gaming Time in the week		Mean Daily Gaming Time in The weekend		Social Media Years		Mean Daily Social Media Usage Time in the week		Mean Daily Social Media Usage Time in the weekend	
N		562		556		557		555		558		545		543	
Mean		29.3		5.62		2.23		3.39		7.06		2.55		3.01	
SD		10.6		4.49		1.82		2.40		4.41		2.16		2.48	
Min		12.0		0.00		0.00		0.00		0.00		0.00		0.00	
Max		68.0		30.0		15.0		18.0		17.0		15.0		16.0

**Table 2:** Participants’ sociodemographic information.

				N		Total N		Proportion	
Gender		Man (cisgender)		283		565		0.501	
		Woman (cisgender)		259		565		0.458	
		Man (transgender)		4		565		0.007	
		Woman (transgender)		1		565		0.002	
		Nonbinary		12		565		0.021	
		Not Listed		3		565		0.005	
		Prefer not to say		3		565		0.005	
Sexual Orientation		Heterosexual-Straight		359		488		0.736	
		Homosexual		36		488		0.074	
		Bisexual		75		488		0.154	
		Asexual		5		488		0.010	
		Other		13		488		0.027	
Ancestry		Aus./Engl.		412		565		0.552	
		Chinese		20		565		0.035	
		German		7		565		0.012	
		Indian		10		565		0.018	
		Other		118		565		0.209	
Occupational Status		Full-Time Employed		271		490		0.553	
		Part-Time Employed		77		490		0.157	
		Student		64		490		0.131	
		Trainee		2		490		0.004	
		Not Currently Working		32		490		0.065	
		On Temporary Leave (Education Leave, Public Service Leave, Training, Maternity Leave)		5		490		0.010	
		Other		39		490		0.080	
Educational Status		Professional Degree (i.e., MD, JD etc. completed)		10		489		0.020	
		PhD Degree (Completed)		17		489		0.035	
		Postgraduate Studies (MSc Completed)		67		489		0.137	
		Undergraduate University Course (completed)		176		489		0.360	
		Intermediate between secondary level and university (e.g., technical training)		97		489		0.198	
		Senior secondary school (Years 11 to 12)		101		489		0.207	
		Secondary school (Years 7 to 10)		9		489		0.018	
		Other		12		489		0.025	
Living arrangement		Family of origin (two parents/partners, only child)		34		564		0.060	
		Family of origin (two parents/partners and siblings)		108		564		0.191	
		Mother (only child, parent divorced-separated-widowed)		19		564		0.034	
		Mother and sibling(s) (parent divorced-separated-widowed)		17		564		0.030	
		Father (only child, parent divorced-separated-widowed)		6		564		0.011	
		Father and sibling(s) (parent divorced-separated-widowed)		5		564		0.009	
		With Partner		149		564		0.264	
		Alone		61		564		0.108	
		With Friend(s)		28		564		0.050	
		Temporary accommodation		4		564		0.007	
		Other		18		564		0.032	
		With Partner and Children		115		564		0.204	
Relationship Status		Single		148		490		0.302	
		In a romantic relationship (A romantic relationship is defined as a romantic commitment of particular intensity between two individuals of the same or the opposite sex (When you like a guy [girl] and he [she] likes you back).		157		490		0.320	
		Engaged		24		490		0.049	
		Married		145		490		0.296	
		Defacto		16		490		0.033	

Using Hill’s (1998) guidelines, a 95 % confidence interval for this sample size (n = 565, z = 1.96) indicates a maximum sampling error of ± 4.12 %. At the initial assessment, missing data ranged from 0.7 % (age data; 4 participants) to 2.83 % (2nd item of the Online Flow Questionnaire; 51 participants), with missing data showing random distribution (MCAR test = 38.4, p = 0.14, indicating no significant deviation from randomness; Little, 1988). To determine the required sample size for the network models, a power analysis was conducted using standard statistical parameters commonly applied in network psychometrics. The calculations were based on the assumption of a medium effect size (d = 0.3), a statistical power of 80 % (1 − β = 0.80), and a significance level of 0.05 (α = 0.05). The analysis was implemented in R using the pwr package (Champely, 2020), which applies Cohen’s d-based power calculations for independent sample comparisons, a standard approach in network-based psychometrics (Epskamp, Borsboom, & Fried, 2018). This methodology was applied to two network structures: (a) a 5-node network, representing flow items, and (b) a 9-node network, consisting of GDT-4 and 5 OFQ items. Both networks were modelled as random graphs using the igraph package (Csardi & Nepusz, 2006), assuming an edge probability of 0.5 to approximate a moderate level of connectivity. Using two-sample *t*-test power estimation, the results indicated that a minimum sample size of 88 participants per group is required for each network to achieve the desired power threshold under the specified conditions. These results are consistent with prior recommendations for power analyses in network estimation (Epskamp et al., 2018, Costantini et al., 2015), which emphasize the importance of adequate sample sizes to ensure stable edge estimation in psychological networks. These provide an empirical basis for sufficient statistical power in network estimation and inference while maintaining methodological robustness. In practice, the required sample size may vary depending on network sparsity, the estimation method (e.g., EBIC-glasso in qgraph, or regularized models in bootnet), and the degree of node interdependence (Williams et al., 2019).

### 2.2 Measures

A questionnaire battery was utilized to measure participant’s socio-demographics (age, gender, education etc.), internet gaming behaviors (involvement in internet gaming, time spent gaming and their history of gaming.), absorbance by gaming activities (Chen et al., 2000) and disordered gaming (Pontes et al., 2021, Pontes and Griffiths, 2015).

#### 2.2.1 Online flow questionnaire (OFQ; Chen et al., 2000)

The OFQ was used to assess engagement with and absorption in online activities. The OFQ consists of five questions based on experiences from the preceding 12 months, exploring four theoretical domains of online flow: Intrinsic motivation, adequate challenge, distorted perceptions of time, and high satisfaction levels. The initial form of the questionnaire involved five flow experience items, followed by a question related to the type of application where the experience was encountered. The items focused on flow experience were originally addressed by a “yes” or “no” answer, with item 5 emphasizing the sense of control the user experiences online (Chen et al., 1999). An example item is: “*When navigating online, have you ever experienced the feeling of ‘time going too fast’?”* The version employed in the present study follows the revised form of the scale,^1^ including only five questions targeting the different aspects of the flow experience (and not the application where this was encountered, as the questionnaire addressed gaming for all participants; Stavropoulos et al., 2022).

Furthermore, in this version, item 5 has been revised to address the sense of disconnection and lack of control a subject may experience while in a state of flow as per recent revisions of the instrument (Hu et al., 2019). Item responses are on a five-point Likert scale from 0 ‘Not at all’ to 4 ‘Absolutely’. High scores on the OFQ reflect a greater amount of online flow experiences, with low scores indicating low experiences of online flow. For this sample, Cronbach’s α and the McDonalds ω internal reliability indices were 0.562 and 0.584, respectively.

The observed Cronbach’s α and McDonald’s ω for the OFQ suggest low internal reliability, which raises concerns regarding the coherence of the construct being measured. Typically, α values above 0.70 are considered acceptable for research purposes, while values below 0.60 indicate potential measurement issues (Nunnally, 1978, Tavakol and Dennick, 2011). Similarly, McDonald’s ω, which accounts for factor loadings, also falls within the low range, reinforcing the need for further scale evaluation (McDonald, 1999).

One possible explanation is multidimensionality, where the scale items may tap into distinct but related subcomponents rather than a single latent construct (Cortina, 1993). This aligns with the PCA results revealed. Additionally, Cronbach’s α is sensitive to the number of items, with shorter scales often yielding lower values, which is the case here (Streiner, 2003, Raykov, 1997, Revelle and Zinbarg, 2009).

To improve reliability, future studies may consider item refinement through differential item functioning (DIF) analysis, the removal of poorly performing items, or the use of alternative reliability indices better suited for short scales. Given these limitations, the interpretation of OFQ-related findings should be approached with caution, as measurement error may influence effect estimates.

#### 2.2.2 Gaming disorder test, 4 items (GDT-4; Pontes et al., 2021)

The GDT-4 is a 4-item scale used to measure gaming disorder symptoms and reflects the WHO criteria for gaming disorder behaviors. Per WHO criteria, questions are focused on experiences within the past 12 months, and measure impaired control over gaming, increased priority of gaming over other activities and continuing to game despite experiencing negative consequences. An example question is: *“I have given increasing priority to gaming over other life interests and daily activities”.* Item responses are recorded on a five-point Likert scale from 0 ‘Never’ to 4 ‘Very often’. High scores on the GDT-4 reflect a higher quantity of disordered gaming experiences, with low scores indicating fewer experiences of disordered gaming. For this sample, Cronbach’s α and the McDonalds ω internal reliability indices were 0.809 and 0.815, respectively.

### 2.3 Procedure

Authorization for the research was granted by the Human Research Ethics Committees at Victoria University [HRE21-044], the Australian State of Victoria's Department of Education and Training [2022_004542], and the Melbourne Archdiocese of Catholic Schools [1179]. The sample was recruited from multiple sources, including tertiary education institutions [e.g., RMIT, Victoria, Melbourne and Deakin Universities], Victorian public and Catholic schools, gaming communities (e.g., Aus Gaymers Network), gaming venues (e.g., Fortress Melbourne), online forums (e.g., AusGamers.com), and YouTube advertisement videos^2^ [e.g., https://www.youtube.com/watch?v=LC1z-7LCArY]. The study invited both adolescents (ages 12 to 18) and adults to participate voluntarily and anonymously. Each prospective participant received a plain-language information statement outlining the study’s purpose, potential risks, and their rights, including the right to withdraw at any time without penalty. For adolescent participants, this information was first reviewed and approved by a parent or guardian through written consent (i.e., a signed information statement), after which the adolescent gave their assent (i.e., a signed information statement). Adults aged 18 and older provided direct consent. Consequently, all participants engaged in the study voluntarily, with a full understanding of its details.

### 2.4 Network analyses

To address the study aims, two network models were computed: Network 1, containing only the primary online flow domains as measured by the OFQ (Chen et al., 2000), and Network 2, the same OFQ (Chen et al., 2000) online flow domains with the addition of disordered gaming behaviors represented by the GDT-4 (Pontes et al., 2021). Edge weights in the network represent the degree of relatedness between the nodes (corresponding to the factors of interest), and zero-order correlations can be utilized to estimate these nodes and edges that comprise the network model. In certain circumstances, the edges between nodes will not account for the relationships with other nodes, exaggerating correlations and producing findings that are deceptive and difficult to understand. To bypass this, network analysis is computed using a regularized partial correlation technique, Least Absolute Shrinkage and Selection Operator (lasso; Tibshirani, 2011). The lasso method operates by reducing small partial correlations to zero, yielding a sparse network representation that displays only the most crucial connections, while typically conferring little risk of false positive edges and thus lending confidence to the reported network structure (Krämer et al., 2009). However, because lasso can produce false negatives, the absence of an edge connecting two nodes should not be taken to imply that there is no relationship.

The network's stability was evaluated by assessing the expected influence centrality stability, edge weight stability, and bridge expected influence stability coefficients against the criteria of ≥ 0.25 as acceptable and ≥ 0.5 as indicating good stability within the model (Epskamp et al., 2018). The coefficients of the network were all deemed as representing good or acceptable stability; expected influence centrality stability coefficient = 0.673, edge stability coefficient = 0.75, and the bridge expected influence coefficient = 0.361.

## 3 Results

The mean and standard deviation scores for the GDT-4 (Pontes et al., 2021) and the OFQ (Chen et al., 2000) were examined and are presented in Table 3.

**Table 3:** Online flow questionnaire and gaming disorder test descriptive statistics (n = 565).

	Valid	Missing	Mean	Std. Deviation	Minimum	Maximum
Impaired Control Over Gaming (GDT 1 Control)	561	4	2.26	0.796	1	4
Increased Priority Given to Gaming (GDT 2 Priority)	561	4	2.41	0.843	1	4
Continuation Despite Negative Consequences (GDT 3 Continuation)	561	4	2.23	0.947	1	4
Experience of Significant Problems in Life (GDT 4 Problems)	560	5	1.78	0.694	1	4
Flow Experience (OFQ 1 Flow)	523	42	2.87	1.35	1	5
Loss of Sense of Time (OFQ 2 Time)	514	51	3.81	1.35	1	5
Enjoyment (OFQ 3 Enjoyment)	553	12	4.15	1.04	1	5
Positive Challenge (OFQ 4 Challenge)	552	13	3.74	1.21	1	5
Loss of Control (OFQ5 Control)	553	12	2.14	1.25	1	5

*Note:***GDT Items**: GDT1 (Control): Impaired control over gaming, indicating difficulty in regulating gaming behavior; GDT2 (Priority): Increased priority given to gaming over other activities and interests; GDT3 (Continuation): Continuation of gaming despite negative consequences; GDT4 (Problems): Experience of significant problems in life due to gaming. **OFQ Items**: OFQ1 (Flow): Overall flow experience, representing a state of deep immersion and engagement in gaming; OFQ2 (Time): Loss of sense of time while gaming, indicating a high level of absorption; OFQ3 (Enjoyment): Enjoyment derived from gaming, reflecting positive emotional responses; OFQ4 (Challenge): Positive challenge experienced during gaming, indicating a balance between skill and difficulty; OFQ5 (Control): Loss of control during gaming, representing a lack of self-regulation.

In regard to the GDT-4, participants reported moderate levels of gaming disorder symptoms, with some variability. The mean scores indicated impaired control over gaming, increased priority given to gaming, continuation despite negative consequences, and significant life problems. The standard deviations suggested variability, with the highest in continuation despite negative consequences and the lowest in significant life problems. This suggests that while some participants experienced significant issues related to gaming, others had fewer problems, indicating a range of gaming disorder severity within the sample. For the OFQ, participants experienced high levels of enjoyment and positive challenge while gaming, with moderate levels of flow and loss of control. The mean scores reflected flow experience, loss of sense of time, enjoyment, positive challenge, and loss of control while gaming. The standard deviations indicated variability, with the highest in flow experience and the lowest in enjoyment. This suggests that while most participants found gaming enjoyable and challenging, their experiences of flow and control varied more widely.

### 3.1 Visualization of the gaming disorder test and online flow questionnaire network

**Fig. 1:**
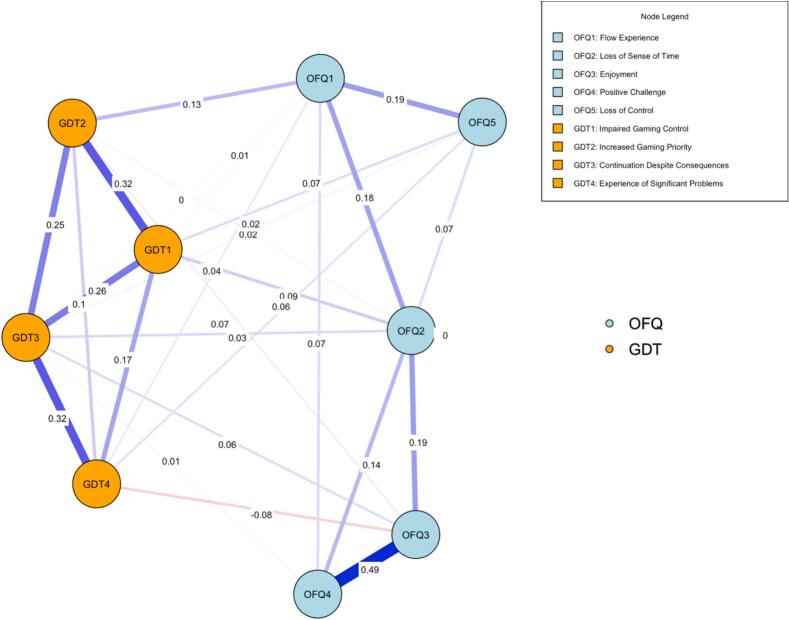
Network of GDT-4 and OFQ items.

A network analysis was conducted with the items for the OFQ and the GDT, resulting in a network of the two constructs presented in Fig. 1. Within the network, blue edges indicate positive relations, and red edges indicate negative relations. Additionally, the edge weights, representing the strength of the relationships between nodes, are visually depicted through variations in the thickness and color saturation of the connecting edges, wherein thicker and more saturated edges correspond to stronger relationships. The distance between nodes is indicative of the relationship between them (i.e., nodes with stronger similarities are closer together).

Congruent with the validated scale, the nodes representing items within the OFQ construct exhibit positive associations amongst themselves, and similarly, the nodes corresponding to GDT demonstrate positive inter-relationships within this network model. Additional edge strengths and centrality statistics for this network are shown in Table 4, Table 5.

**Table 4:** Gaming disorder test and online flow questionnaire edge weights matrix.

Variable	GDT1	GDT2	GDT3	GDT4	OFQ1	OFQ2	OFQ3	OFQ4	OFQ5
GDT1	0	0.32	0.25	0.17	0.01	0.09	0.00	0.00	0.06
GDT2	0.32	0	0.24	0.09	0.13	0.01	0.03	0.00	0.00
GDT3	0.25	0.24	0	0.32	0.00*	0.07	0.06	0.01	0.01
GDT4	0.17	0.09	0.32	0	0.04	0.00	−0.08	0.00	0.05
OFQ1	0.01	0.13	0.00*	0.04	0	0.18	0.00	0.07	0.19
OFQ2	0.09	0.01	0.07	0.00	0.18	0	0.19	0.13	0.07
OFQ3	0.00	0.03	0.06	−0.08	0.00	0.19	0	0.49	−0.00**
OFQ4	0.00	0.00	0.01	0.00	0.07	0.13	0.49	0	0.00
OFQ5	0.06	0.00	0.01	0.05	0.19	0.07	−0.00**	0.00	0

Note: * < −0.003; ** < −0.00001.

**Table 5:** Gaming disorder test and online flow questionnaire network centrality measures.

Variable	Betweenness	Closeness	Strength	Expected Influence
GDT1	−0.64	0.76	0.88	1.06
GDT2	0.32	0.39	0.43	0.63
GDT3	−0.32	0.43	1.31	1.48
GDT4	−0.32	−0.09	0.00	−0.70
Flow1	1.62	1.23	−0.78	−0.56
Flow2	1.62	0.79	−0.03	0.16
Flow3	−0.32	−0.44	0.53	−0.18
Flow4	−0.97	−1.44	−0.28	−0.07
Flow5	−0.97	−1.62	−2.07	−1.83

### 3.2 Gaming disorder and online flow network characteristics

As shown in Table 3 and Fig. 1, the most significant edges between nodes of the flow construct are represented in descending strength; with the strongest edge weights seen between items OFQ3 (Enjoyment) and OFQ4 (Challenge), items OFQ1 (Flow) and OFQ5 (Control), items OFQ2 (Time) and OFQ3 (Enjoyment). Of note, the edges between nodes of the Online flow construct that presented 0 significant connections were OFQ items 1 and 3, 3 and 5, and 4 and 5. The lasso regularization method may produce false negatives. Thus the absence of an edge should not be interpreted as a definitive lack of relationship between these items; instead, it suggests that any existing relationship may be weak or statistically non-significant in this context (Krämer et al., 2009).

**Fig. 2:**
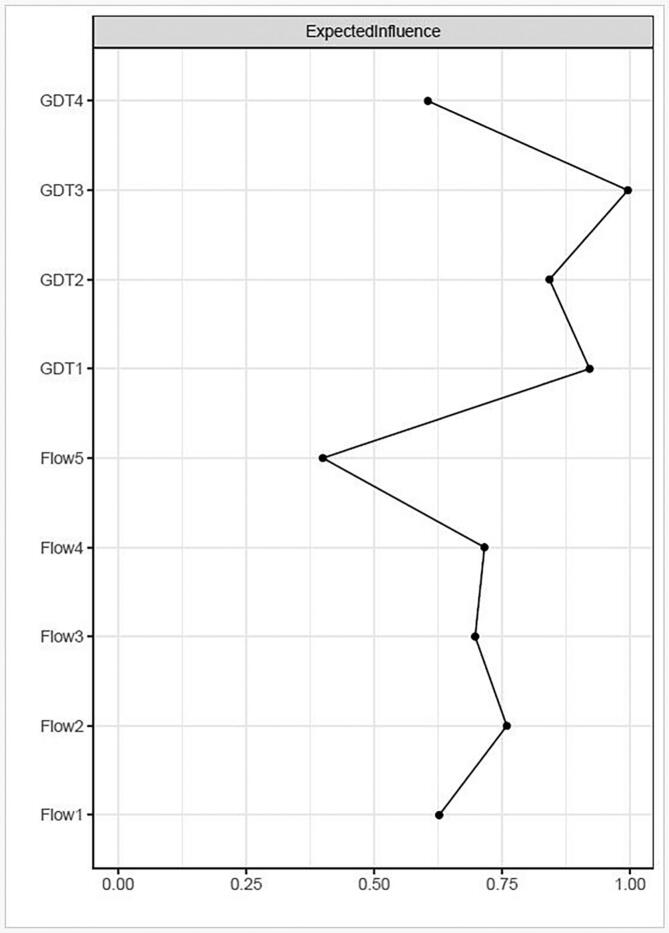
Expected influence across nodes.

**Fig. 3:**
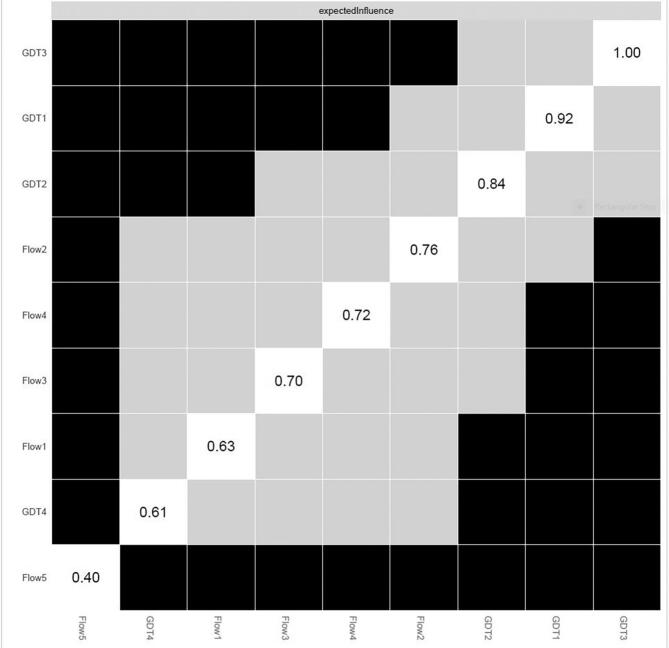
Centrality difference tests for expected influence of nodes.

Table 2 and Fig. 1 depict the edges of greatest significance between nodes for the GDT construct; the most significant of these are the edges between GDT1 (Control) and GDT3 (Continuation), GDT3 (Continuation) and GDT4 (Problems) and finally, GDT1 (Control) and GDT2 (Priority). Fig. 2, Fig. 3 represent the expected influence centrality of nodes across the network and the centrality difference test of which nodes have the greatest influence over others.

Within this combined network, both the OFQ construct, and GDT symptoms are centered in clusters; OFQ items 2, 4, 3 and 1 in descending order of expected influence; and GDT 3, 1 and 2, respectively. The most important node is recognized as the item or symptom (node) within a Network that is most central to the construct/concept and therefore contains the greatest number of and strongest connections to other nodes. Within the online flow construct, this was OFQ2 (Time), and within the Gaming Disorder symptoms it was GDT3 (Continuation).

**Fig. 4:**
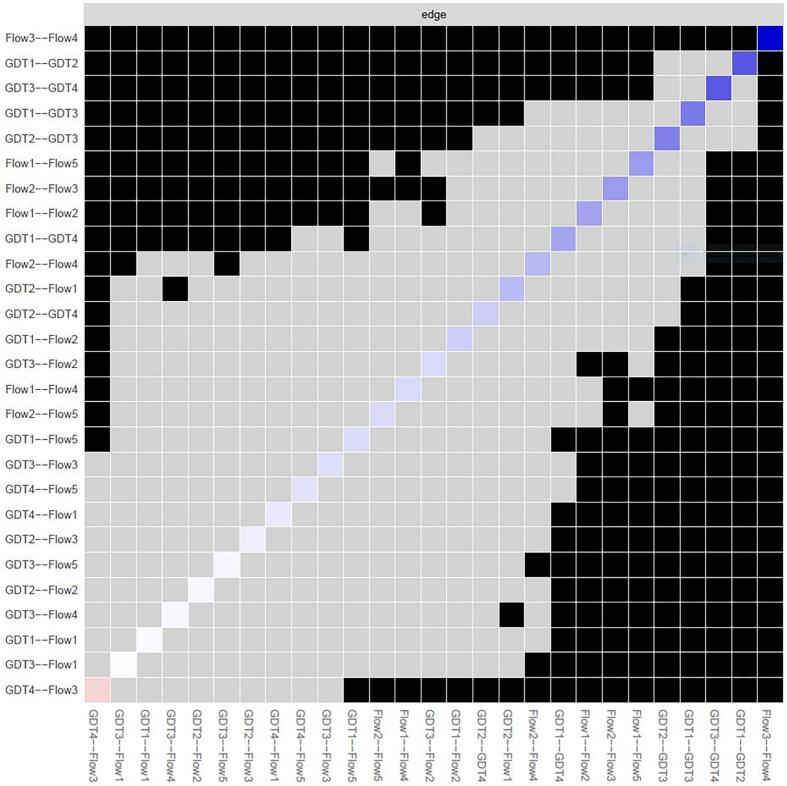
Edge difference test for edges between nodes.

To assess the statistical significance of differences in association strengths between nodes, edge weight difference tests were performed, the results of which are depicted in Fig. 4, wherein the edge connecting nodes Flow 3 (Enjoyment), and Flow 4 (Challenge) exhibits the largest weight (represented by the darkest blue coloring). Notably, the edge connecting nodes Flow 3 (Enjoyment) and GDT4 (Problems) is depicted in an orange/red color, indicating a negative association.

### 3.3 Bridge characteristics

**Fig. 5:**
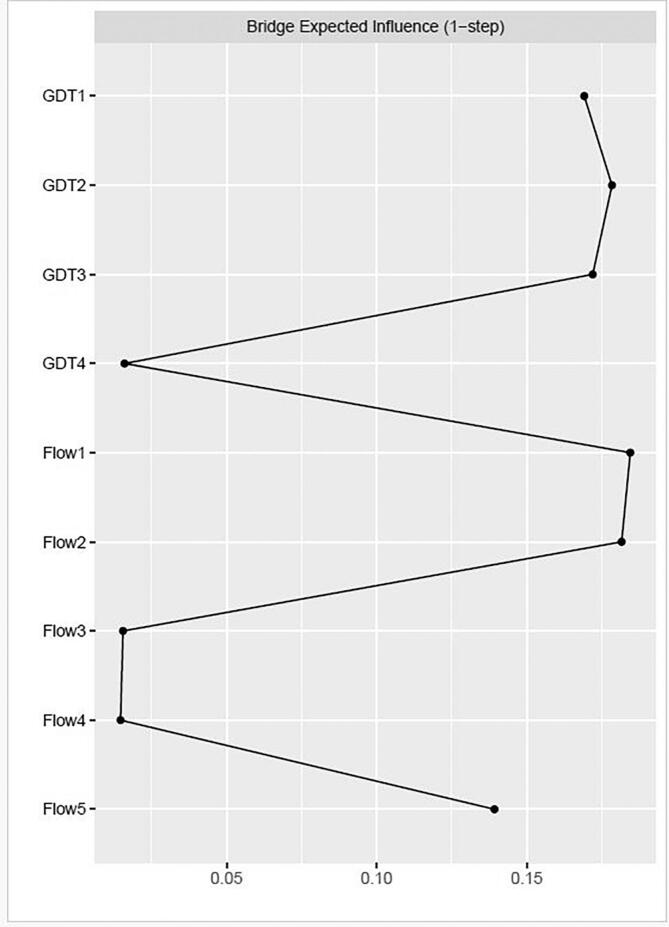
Bridge expected influence between OFQ and GDT.

Regarding bridge characteristics, Fig. 5 presents the bridge expected influence between the OFQ and GDT constructs. Within the online flow construct, items 1 (Flow) and 2 (Time) exhibited the highest levels of bridge expected influence, acting as primary bridge nodes. The OFQ5 (Control) demonstrated a secondary level of bridge expected influence. For the gaming disorder symptomatology, GDT1 (Control), 2 (Priority), and 3 (Continuation) displayed comparable and relatively high levels of bridge expected influence, whereas GDT4 (Problems) exhibited negligible bridge expected influence as a node connecting the two constructs.

## 4 Discussion

The present network analysis findings provide salient insights into the key components underpinning and constituting online flow state experiences within gaming contexts. Advanced network modelling techniques were applied to discern the central and peripheral features underlying online flow occurrences and examine their associations with disordered gaming patterns. Notably, the network revealed a remarkable coherence, with close centrality observed for all items except “*Experience of Significant Problems in Life*” (GDT4). Enjoyment and positive challenge emerged as particularly central elements, indicating their significant roles in shaping flow experiences. This may suggest an intricate interconnectedness among flow components, emphasizing the holistic nature of flow experiences within gaming.

The results demonstrated that the experience of a ‘*loss of sense of time*’, defined as the altered or distorted experience of time passing (Csikszentmihalyi, 1990), was the most central component underlying the online flow experience. This node had the strongest connections to other flow features and was closely followed by the positive challenge and enjoyment combining to represent the central structure of the online flow construct. The strongest individual connections (i.e., edge weights) were seen between enjoyment and positive challenge, which additionally had the strongest connection across the entire network, suggesting they are strongly interconnected and aligning with conceptualizations of flow involving a dynamic skill-challenge balance (Csikszentmihalyi, 1990).

The nodes forming a bridge between the online flow construct and IGD construct showed the greatest influence (in order of strength) on the model between a three-node core of GDT 3 (Continuation), 1 (Control) and 2 (Priority) positively related to the OFQ’s 1 (Flow) and 2 (Time) nodes, with 5 (Control) as a secondary bridging pathway. An additional bridge of interest, though with the weakest edge weight within the entire model, was the negative association between ‘enjoyment within online flow’ and ‘significant life problems’ within the GDT. This connection may be potentially explained by some level of the addictive process, whereby the user no longer enjoys the activity but continues to engage to the point of feeling their behavior is problematic (Gros et al., 2020), further feeding into the third highest edge weight connection between GDT4 (Problems) and GDT3 (Continuation). Moreover, the absence of direct connections between nodes representing flow experience and those corresponding to challenge and enjoyment may indicate that enjoyment and the experience of flow reflect distinct facets or pathways within online gaming experiences, rather than components of a singular, linear experience (Kiatsakared & Chen, 2022).

GDT1 (Control) and OFQ2 (Time) were also strongly connected, reflecting absorptive states with disrupted awareness (Jennett et al., 2008). Conversely, GDT2 (Priority) and GDT3 (Continuation) demonstrated moderate associations with online flow components, indicating a nuanced relationship between disordered gaming symptoms and flow experiences. Although GDT1 (Priority) demonstrated the strongest individual connection to the flow experience among the gaming disorder nodes, the three-core nodes of gaming disorder—priority, continuation, and control—showed connections to the components of flow related to time distortion and loss of control. However, these relationships were notably weaker than anticipated. This finding contrasts with previous research which has typically reported strong positive relationships between flow states and disordered gaming symptoms (Nuyens et al., 2019). Indeed, these findings diverge from some prior gaming research utilizing composite flow scales that obscure potential distinctions between facets like focused immersion, enjoyment, and control (Wu et al., 2011).

The network analysis within the present study highlights specific online flow components and dynamics. The OFQ5 (Control) was most peripheral within flow, whereas OFQ4 (Challenge) and OFQ2 (Time) were central. This challenges notions that control loss is inherent to flow and suggests it may reflect a distinctly different pathway or activation to achieve an online flow state (Kiatsakared & Chen, 2022). The weak associations observed between nodes representing disordered gaming and features of flow are also noteworthy, underscoring that these constructs are distinct from one another, yet linked through a shared pathway of heightened engagement (Zhang et al., 2022). These findings suggest that while online flow and disordered gaming relate to one another to some extent, they are ultimately distinct phenomena involving several disparate underlying mechanisms. Such insights have implications for intentionally optimizing the flow state in serious games to enhance learning and engagement (Buzady et al., 2022), while concurrently mitigating the risks associated with immersive states linked to disordered gaming symptomatology (Larche & Dixon, 2021). Additionally, tailoring in-game challenges to progressively match rising player skill levels may help sustain enjoyable flow experiences (Antons et al., 2023, Buzady et al., 2022).

The current findings also highlight key differences between online flow states and disordered gaming patterns. Specifically, the network analysis revealed several weak associations between disordered gaming symptoms and online flow components. This contrasts with past research that has found robust correlations between flow experiences and disordered gaming behaviors (i.e., dark flow; Hu et al., 2019, Zhang et al., 2022). Once again, the weak ties observed in the present study suggest that while similar in the effects of control loss on individuals, online flow and disordered gaming reflect distinct phenomena with varying cognitive and neurobiological states, each with distinct implications for control and engagement (Dong et al., 2017, Lee et al., 2020, Nuyens et al., 2019).

Notably, the flow components OFQ3 (Enjoyment) and OFQ4 (Challenge) were only weakly tied to disordered gaming symptoms in the network model. This implies enhancing enjoyment and positive challenge need not increase disordered gaming risks. These elements can instead be leveraged to intentionally promote adaptive flow states that boost learning, engagement, and wellbeing through serious gaming applications (Buzady et al., 2022, Zairi et al., 2022). The network analysis instead revealed bridges between flow experience, time distortion, and disordered gaming symptoms, suggesting that prolonged, intensely focused gaming may increase vulnerabilities to problematic gaming in some instances, particularly when it triggers escapism (Hu et al., 2021). As such, facilitating self-monitoring and moderation of periods of absorbed gaming (e.g., setting alarms to take regular breaks) may help minimize risks of developing disordered patterns for some gamers (Colder Carras et al., 2020). Overall, the network analysis indicates online flow and disordered gaming are related but distinct experiences involving different mechanisms. Understanding these nuances has important implications for selectively promoting adaptive flow while also identifying and mitigating problematic gaming risks.

### 4.1 Limitations

While providing important insights, the current study has some limitations that should be acknowledged. Firstly, the reliance on self-report measures for assessing online flow and disordered gaming symptoms presents issues with recall biases and social desirability. Future studies would benefit from utilizing multiple methods, including both psychometric scales and objective behavioral metrics of gaming use (Nacke et al., 2011). For example, physiological measurements of arousal and eye-tracking technology could help objectively quantify states of immersion during gaming (Al-Hargan et al., 2017). It should be noted that item 5 of the OFQ was modified from its original form which measured sense of control, to instead assess experiences of disconnection and loss of control during flow states. While this adaptation aligns with recent theoretical developments (Hu et al., 2019), this modification should be considered when comparing findings with studies using the original version. It should also be noted that, for this sample, the Cronbach’s α and McDonald's ω internal reliability coefficients indicate potential issues with the robustness of the instrument and the validity of the results. It is important to consider these limitations when interpreting the findings. Additionally, the cross-sectional nature of this study precludes determining causality regarding the interplay between online flow, enjoyment, and problematic gaming patterns. Longitudinal designs tracking gamers over time would better clarify causal pathways. Experimental manipulations inducing different components of flow states could also prove insightful for isolating their effects on gaming experiences and problematic behaviors (Nacke et al., 2011). The sample recruited here was predominantly young adult Australians, thus findings may not generalize to wider gaming populations; as such, collecting larger global samples would increase generalizability. Additionally, the present study collected data from role-playing-gamers, which may further impact its generalizability to all gamers. Future studies should aim to collect and analyze data on game types to address this limitation. Finally, network analysis findings are dependent on the measures used to construct the models (Krämer et al., 2009), thus incorporating additional behavioral indicators could provide a more comprehensive mapping of online flow and disordered gaming compositions.

Moving forward, research leveraging intensive longitudinal methods, objective behavioral metrics, neurophysiological assessments, and experimental manipulations are needed to substantiate the network dynamics identified here. Replicating this study’s modelling approach amongst clinically diagnosed samples of disordered gamers would also be beneficial to determine differences between community-based and clinical populations. There are also opportunities for applying network analysis to discern nuances within related phenomena like Esports engagement, virtual reality gaming, and live-streaming interactions. Such research can refine conceptualizations and assessments of both adaptive and maladaptive gaming patterns.

### 4.2 Implications & conclusions

The present study offers several salient implications for research and clinical contexts regarding online gaming experiences. The network analysis approach implemented here provides a nuanced discernment of key components underlying online flow states versus disordered gaming patterns. This advances conceptual clarity and assessment capabilities beyond conventional correlational research. The findings reveal that while both online flow and disordered gaming patterns show connections within the network, they differ significantly in their centrality, reflecting their varying levels of importance within the network. Loss of control appears to be peripheral to the broader flow structure, with a moderate connection to the construct. While potentially linked to other flow features, its role in the flow state experience remains tentative and requires further investigation. Consequently, deliberately fostering enjoyment and other positive aspects of flow whilst providing awareness of one’s control loss experiences appears prudent for promoting adaptive engagement in serious games and minimizing disordered gaming risks. Indeed, tailoring skill-challenge balances to sustain flow also holds promise as an intervention and game design strategy (Dias et al., 2018). Crucially, loss of sense of time emerged as a potential bridge between online flow and disordered gaming, suggesting that targeting control restoration (e.g., through setting alarms and reminders to take breaks) around continuation in gaming despite the consequences could be effective in mitigating problematic gaming patterns (Colder Carras et al., 2020). These findings align with clinical models that place loss of control as central to gaming disorder and may enhance current approaches to assessment and diagnosis. Overall, the present paper substantiates important distinctions between online flow and disordered gaming in terms of their underlying behavioral compositions.

**Informed consent was obtained from all participants included in the study**

All procedures in studies involving human participants were performed in accordance with the ethical standards of the Victoria University Human Research Ethics Committee [HRE21-044], the Department of Education and Training of The Victorian State Government, Australia [2022_004542] and the Melbourne Archdiocese of Catholic Schools [1179].

**Funding**

While working on the manuscript, Dr. Vasileios Stavropoulos was supported by a grant from the 10.13039/501100000923Australian Research Council, Discovery Early Career Researcher Award, 2021, number DE210101107.

**Ethical standards – animal rights**

All procedures performed in the study involving human participants were in accordance with the ethical standards of the institutional and/or national research committee and with the 1964 Helsinki declaration and its later amendments or comparable ethical standards. This article does not contain any studies with animals performed by any of the authors.

**Confirmation statement**

Authors confirm that this paper has not been either previously published or submitted simultaneously for publication elsewhere.

### CRediT authorship contribution statement

**Tyrone L. Burleigh:** Writing – review & editing, Writing – original draft, Conceptualization, Data curation, Formal analysis, Methodology, Validation, Visualization. **Trent Footitt:** Writing – review & editing, Writing – original draft, Methodology, Investigation, Formal analysis. **Michelle Colder Carras:** Writing – review & editing. **Connor Conkey-Morrison:** Writing – review & editing, Writing – original draft. **Dylan R. Poulus:** Writing – review & editing. **Vasileios Stavropoulos:** Writing – review & editing, Supervision, Methodology, Investigation, Formal analysis, Data curation, Validation, Visualization.

## Declaration of competing interest

The authors declare that they have no known competing financial interests or personal relationships that could have appeared to influence the work reported in this paper.

## Data Availability

Files have been included as supplementary materials

## Footnotes

1 A PCA analysis with Oblimin rotation revealed two factors: Items 2, 3, and 4 grouped together, suggesting an egosyntonic flow experience, while Items 1 and 5 formed a separate factor, likely reflecting egodystonic flow experiences. Parallel analysis confirmed this two-factor structure, supporting the validity of the overall flow score. The fit indices, such as the eigenvalues (1.85 and 1.41) explaining 65% of the variance, and an RMSR of 0.14, indicate a reasonable fit. A validated tool is essential and should be considered for future research to ensure robustness and reliability. See appendices for further information.

2 YouTube videos created by the research team were shared via social media links and QR codes with recruitment partners (schools, etc.) to recruit participants. It is important to note this wasn’t targeted advertising; it was a link included in communications to these partners.
