# Supplementary material for: Flowing in the net of disordered gaming: A network analysis approach
Source: Addict Behav Rep. 2025 Apr 13;21:100606. doi: 10.1016/j.abrep.2025.100606 (PMC12032186; doi:10.1016/j.abrep.2025.100606)
Supplement: Supplementary Data 1 [file mmc1.pdf]

````
---
title: "Flow + GDT Network Analysis"
author: "X"
output:
  pdf_document: default
  html_document: default
---

```{r setup, include=FALSE}
knitr::opts_chunk$set(echo = TRUE)
knitr::opts_chunk$set(message = FALSE)
knitr::opts_chunk$set(warning = FALSE)
```

Setting up packages

```{r}
library(bootnet)
library(networktools)
library(NetworkComparisonTest)
library(qgraph)
library(psych)
library(psychotools)
library(tidyverse)
library(semTools)
library(semPlot)
library(haven)
library(readr)
library(ggplot2)
library(igraph)
library(Matrix)
library(knitr)
```

Loading Data

```{r}
knitr::opts_chunk$set(echo = TRUE)
data<-read_sav("Location of data")
```

Selecting Variables and Assigning Names

```{r}
myvars<-c("FlowQ1_W1", "FlowQ2_W1", "FlowQ3_W1", "FlowQ4_W1", "FlowQ5_W1", "GD_Q1_W1",
"GD_Q2_W1", "GD_Q3_W1", "GD_Q4_W1")

names <- c("OFQ1", "OFQ2", "OFQ3", "OFQ4", "OFQ5", "GDT1", "GDT2", "GDT3", "GDT4")
names1 <- c("Flow Experience", "Loss of Sense of Time", "Enjoyment", "Positive
Challenge", "Loss of Control", "Impaired Gaming Control", "Increased Gaming Priority",
"Continuation Despite Consequences", "Experience of Significant Problems")
FlowGDTNet<-data[myvars]
#Here, myvars contains the names of the variables to be included in the network
analysis. names and names1 provide short and descriptive names for these variables,
respectively. The FlowGDTNet data frame is created by selecting the specified variables
from the original data.
```

Estimating Network- Using defaults

```{r}
network1 <- estimateNetwork(FlowGDTNet, default="EBICglasso")
#The estimateNetwork function is used to estimate the network structure of the selected
variables using the Extended Bayesian Information Criterion Graphical Lasso
(EBICglasso) method.
```

Group Nodes
````

```{r}
groups1=list("OFQ"=c(1:5), "GDT"=c(6:9))
#The groups1 list is created to group the nodes into two categories: OFQ (Flow Experience) and GDT (Gaming Disorder).
```

Bootstrapping Estimate

```{r}
b1 <- bootnet(network1, boots=1000, statistics=c("strength", "expectedInfluence", "betweenness", "closeness", "edge"))
b2 <- bootnet(network1, boots=1000, type="case", statistics=c("strength", "expectedInfluence", "betweenness", "closeness", "edge"))
#The bootnet function is used to perform bootstrapping on the estimated network to assess the stability of the network metrics. The function runs 1000 bootstrap samples and calculates various statistics, including strength, expected influence, betweenness, closeness, and edge weights.
```

Centrality Stability Coefficient

```{r}
corStability(b2)
#This function calculates the centrality stability coefficient for the bootstrapped network b2. It assesses the stability of centrality indices across bootstrap samples.
```

Plot Centrality Stability Graphs

```{r}

plot(b2)
dev.off()


plot(b2, "ExpectedInfluence")
dev.off()


plot(b2, "betweenness")
dev.off()


plot(b2, "closeness")
dev.off()
#These commands generate and save plots for the centrality stability of the bootstrapped network b2. The plots include overall centrality, expected influence, betweenness, and closeness.
```

Strength Centrality Diff Test

```{r}

plot(b1, "strength", order="sample", labels=TRUE)
dev.off()
#This command plots the strength centrality difference test for the bootstrapped network b1, ordering the nodes by sample and including labels.
```

Expected Inflience Centrality Diff test

```{r}

plot(b1, "expectedInfluence", order="sample", labels=TRUE)
dev.off()
#This command plots the expected influence centrality difference test for the bootstrapped network b1, ordering the nodes by sample and including labels.
```

Edge Stability Graph

```{r}

plot(b1, labels = FALSE, order = "sample")
dev.off()
#This command generates and saves an edge stability graph for the bootstrapped network b1, ordering the nodes by sample without labels.
```

Edge Weights Stability

```{r}

plot(b1, "edge", plot="difference", onlyNonZero=TRUE, order = "sample")
dev.off()
#This command plots the stability of edge weights for the bootstrapped network b1, showing only non-zero differences and ordering the nodes by sample.
```

Strength/Expected Influence Centrality Plots

```{r}

c1 <- centralityPlot(network1, include = c("ExpectedInfluence"), orderBy ="default")
dev.off()


c2 <- centralityPlot(network1, include = c("Betweenness", "Closeness"), orderBy
="default")
dev.off()
#These commands generate and save centrality plots for the original network network1, including expected influence, betweenness, and closeness, ordered by default.
```

Edge Strength/Correlations

```{r}
edges1<-getWmat(network1)
write.csv(edges1, "edges1.csv")
#This command extracts the edge weights matrix from the original network network1 and saves it as a CSV file named edges1.csv.
```

Inspect Centrality Indices

```{r}

Centrality1 <- centralityTable(network1)
write.csv(Centrality1, "Centrality1.csv")
view(Centrality1)
#These commands create a table of centrality indices for the original network network1, save it as a CSV file named Centrality1.csv, and display the table.
```

Network Plot With Groups

```{r}
jpeg("plot1.jpg")
pdf("Plot1.pdf")
plot1 <- plot(network1, layout="spring", vsize=6, border.color="black", groups=groups1,
color=c('lightblue', 'orange'),labels=names)
dev.off()


plot1 <- plot(network1, layout="spring", vsize=6, border.color="black", groups=groups1,
color=c('lightblue', 'orange'))
dev.off()
```

```
plot1 <- plot(network1, layout="spring", vsize=6, border.color="black", groups=groups1, color=c('lightblue', 'orange'),labels=names1)
dev.off()
#These commands generate and save network plots for the original network network1 with different layouts and groupings, saving the plots as JPEG and PDF files.
```

Estimate Bridge Values

```{r}

bridge1<-bridge(plot1, communities=c('1', '1', '1', '1', '1', '2', '2', '2', '2'), useCommunities = "all", directed = NULL, nodes = NULL)
view(bridge1)
#This command estimates bridge values for the network plot1, identifying nodes that act as bridges between communities.
```

Bridge Strength Plot

```{r}
pdf("bridgecentrality1.pdf", width = 5)
plot(bridge1, include = "Bridge Strength")
dev.off()
#This command generates and saves a plot of bridge strength for the estimated bridge values bridge1, saving the plot as a PDF file.
```

Flow + GDT Network Analysis

[redacted]

Setting up packages

```
library(bootnet)
library(networktools)
library(NetworkComparisonTest)
library(qgraph)
library(psych)
library(psychotools)
library(tidyverse)
library(semTools)
library(semPlot)
library(haven)
library(readr)
library(ggplot2)
library(igraph)
library(Matrix)
library(knitr)
```

Loading Data

```
knitr::opts_chunk$set(echo = TRUE)
data<-read_sav("[redacted]
```

Selecting Variables and Assigning Names

```
myvars<-c("FlowQ1_W1", "FlowQ2_W1", "FlowQ3_W1", "FlowQ4_W1", "FlowQ5_W1", "GD_Q1_W1", "GD_Q2
_W1", "GD_Q3_W1", "GD_Q4_W1")

names <- c("OFQ1", "OFQ2", "OFQ3", "OFQ4", "OFQ5", "GDT1", "GDT2", "GDT3", "GDT4")
names1 <- c("Flow Experience", "Loss of Sense of Time", "Enjoyment", "Positive Challenge", "L
oss of Control", "Impaired Gaming Control", "Increased Gaming Priority", "Continuation Despit
e Consequences", "Experience of Significant Problems")
FlowGDTNet<-data[myvars]
```

Estimating Network- Using defaults

```
network1 <- estimateNetwork(FlowGDTNet, default="EBICglasso")
```

Group Nodes

```
groups1=list("OFQ"=c(1:5), "GDT"=c(6:9))
```

Bootstrapping Estimate

```
b1 <- bootnet(network1, boots=1000, statistics=c("strength", "expectedInfluence", "betweennes
s", "closeness", "edge"))
```

```
##
  |
  |                                                                      |   0%
  |
  |                                                                      |   1%
  |
  |=                                                                     |   1%
  |
  |=                                                                     |   2%
  |
  |==                                                                    |   2%
  |
  |==                                                                    |   3%
  |
  |==                                                                    |   4%
  |
  |===                                                                   |   4%
  |
  |===                                                                   |   5%
  |
  |====                                                                  |   5%
  |
  |====                                                                  |   6%
  |
  |=====                                                                 |   6%
  |
  |=====                                                                 |   7%
  |
  |=====                                                                 |   8%
  |
  |======                                                                |   8%
  |
  |======                                                                |   9%
  |
  |=======                                                               |   9%
  |
  |=======                                                               |  10%
  |
  |=======                                                               |  11%
  |
  |========                                                              |  11%
  |
  |========                                                              |  12%
  |
  |=========                                                             |  12%
  |
  |=========                                                             |  13%
  |
  |=========                                                             |  14%
  |
  |==========                                                            |  14%
```

```
|
|==========                                                            |  15%
|
|===========                                                           |  15%
|
|===========                                                           |  16%
|
|============                                                          |  16%
|
|============                                                          |  17%
|
|============                                                          |  18%
|
|=============                                                         |  18%
|
|=============                                                         |  19%
|
|==============                                                        |  19%
|
|==============                                                        |  20%
|
|==============                                                        |  21%
|
|===============                                                       |  21%
|
|===============                                                       |  22%
|
|================                                                      |  22%
|
|================                                                      |  23%
|
|================                                                      |  24%
|
|=================                                                     |  24%
|
|=================                                                     |  25%
|
|==================                                                    |  25%
|
|==================                                                    |  26%
|
|===================                                                   |  26%
|
|===================                                                   |  27%
|
|===================                                                   |  28%
|
|====================                                                  |  28%
|
|====================                                                  |  29%
|
```

```
|=====================                                                 |  29%
|
|=====================                                                 |  30%
|
|=====================                                                 |  31%
|
|======================                                                |  31%
|
|======================                                                |  32%
|
|=======================                                               |  32%
|
|=======================                                               |  33%
|
|=======================                                               |  34%
|
|========================                                              |  34%
|
|========================                                              |  35%
|
|=========================                                             |  35%
|
|=========================                                             |  36%
|
|==========================                                            |  36%
|
|==========================                                            |  37%
|
|==========================                                            |  38%
|
|===========================                                           |  38%
|
|===========================                                           |  39%
|
|============================                                          |  39%
|
|============================                                          |  40%
|
|============================                                          |  41%
|
|=============================                                         |  41%
|
|=============================                                         |  42%
|
|==============================                                        |  42%
|
|==============================                                        |  43%
|
|==============================                                        |  44%
|
|===============================                                       |  44%
```

```
  |
  |===============================                                      |  45%
  |
  |================================                                     |  45%
  |
  |================================                                     |  46%
  |
  |=================================                                    |  46%
  |
  |=================================                                    |  47%
  |
  |=================================                                    |  48%
  |
  |==================================                                   |  48%
  |
  |==================================                                   |  49%
  |
  |===================================                                  |  49%
  |
  |===================================                                  |  50%
  |
  |===================================                                  |  51%
  |
  |====================================                                 |  51%
  |
  |====================================                                 |  52%
  |
  |=====================================                                |  52%
  |
  |=====================================                                |  53%
  |
  |=====================================                                |  54%
  |
  |======================================                               |  54%
  |
  |======================================                               |  55%
  |
  |=======================================                              |  55%
  |
  |=======================================                              |  56%
  |
  |========================================                             |  56%
  |
  |========================================                             |  57%
  |
  |========================================                             |  58%
  |
  |=========================================                            |  58%
  |
  |=========================================                            |  59%
  |
```

```
|==========================================                            |  59%
|
|==========================================                            |  60%
|
|==========================================                            |  61%
|
|===========================================                           |  61%
|
|===========================================                           |  62%
|
|============================================                          |  62%
|
|============================================                          |  63%
|
|============================================                          |  64%
|
|=============================================                         |  64%
|
|=============================================                         |  65%
|
|==============================================                        |  65%
|
|==============================================                        |  66%
|
|===============================================                       |  66%
|
|===============================================                       |  67%
|
|===============================================                       |  68%
|
|================================================                      |  68%
|
|================================================                      |  69%
|
|=================================================                     |  69%
|
|=================================================                     |  70%
|
|=================================================                     |  71%
|
|==================================================                    |  71%
|
|==================================================                    |  72%
|
|===================================================                   |  72%
|
|===================================================                   |  73%
|
|===================================================                   |  74%
|
|====================================================                  |  74%
```

```
|
|====================================================                  |  75%
|
|=====================================================                 |  75%
|
|=====================================================                 |  76%
|
|======================================================                |  76%
|
|======================================================                |  77%
|
|======================================================                |  78%
|
|=======================================================               |  78%
|
|=======================================================               |  79%
|
|========================================================              |  79%
|
|========================================================              |  80%
|
|========================================================              |  81%
|
|=========================================================             |  81%
|
|=========================================================             |  82%
|
|==========================================================            |  82%
|
|==========================================================            |  83%
|
|==========================================================            |  84%
|
|===========================================================           |  84%
|
|===========================================================           |  85%
|
|============================================================          |  85%
|
|============================================================          |  86%
|
|=============================================================         |  86%
|
|=============================================================         |  87%
|
|=============================================================         |  88%
|
|==============================================================        |  88%
|
|==============================================================        |  89%
|
```

```
  |===============================================================       |  89%
  |
  |===============================================================       |  90%
  |
  |===============================================================       |  91%
  |
  |================================================================      |  91%
  |
  |================================================================      |  92%
  |
  |=================================================================     |  92%
  |
  |=================================================================     |  93%
  |
  |=================================================================     |  94%
  |
  |==================================================================    |  94%
  |
  |==================================================================    |  95%
  |
  |===================================================================   |  95%
  |
  |===================================================================   |  96%
  |
  |====================================================================  |  96%
  |
  |====================================================================  |  97%
  |
  |====================================================================  |  98%
  |
  |===================================================================== |  98%
  |
  |===================================================================== |  99%
  |
  |======================================================================|  99%
  |
  |======================================================================| 100%
## 
  |
  |                                                                      |   0%
  |
  |                                                                      |   1%
  |
  |=                                                                     |   1%
  |
  |=                                                                     |   2%
  |
  |==                                                                    |   2%
  |
  |==                                                                    |   3%
  |
```

```
|==                                                                    |   4%
|
|===                                                                   |   4%
|
|===                                                                   |   5%
|
|====                                                                  |   5%
|
|====                                                                  |   6%
|
|=====                                                                 |   6%
|
|=====                                                                 |   7%
|
|=====                                                                 |   8%
|
|======                                                                |   8%
|
|======                                                                |   9%
|
|=======                                                               |   9%
|
|=======                                                               |  10%
|
|=======                                                               |  11%
|
|========                                                              |  11%
|
|========                                                              |  12%
|
|=========                                                             |  12%
|
|=========                                                             |  13%
|
|=========                                                             |  14%
|
|==========                                                            |  14%
|
|==========                                                            |  15%
|
|===========                                                           |  15%
|
|===========                                                           |  16%
|
|============                                                          |  16%
|
|============                                                          |  17%
|
|============                                                          |  18%
|
|=============                                                         |  18%
```

```
|
|=============                                                          |  19%
|
|==============                                                         |  19%
|
|==============                                                         |  20%
|
|==============                                                         |  21%
|
|===============                                                        |  21%
|
|===============                                                        |  22%
|
|================                                                       |  22%
|
|================                                                       |  23%
|
|================                                                       |  24%
|
|=================                                                      |  24%
|
|=================                                                      |  25%
|
|==================                                                     |  25%
|
|==================                                                     |  26%
|
|===================                                                    |  26%
|
|===================                                                    |  27%
|
|===================                                                    |  28%
|
|====================                                                   |  28%
|
|====================                                                   |  29%
|
|=====================                                                  |  29%
|
|=====================                                                  |  30%
|
|=====================                                                  |  31%
|
|======================                                                 |  31%
|
|======================                                                 |  32%
|
|=======================                                                |  32%
|
|=======================                                                |  33%
|
```

```
|=======================                                               |  34%
|
|========================                                              |  34%
|
|========================                                              |  35%
|
|=========================                                             |  35%
|
|=========================                                             |  36%
|
|==========================                                            |  36%
|
|==========================                                            |  37%
|
|==========================                                            |  38%
|
|===========================                                           |  38%
|
|===========================                                           |  39%
|
|============================                                          |  39%
|
|============================                                          |  40%
|
|============================                                          |  41%
|
|=============================                                         |  41%
|
|=============================                                         |  42%
|
|==============================                                        |  42%
|
|==============================                                        |  43%
|
|==============================                                        |  44%
|
|===============================                                       |  44%
|
|===============================                                       |  45%
|
|================================                                      |  45%
|
|================================                                      |  46%
|
|=================================                                     |  46%
|
|=================================                                     |  47%
|
|=================================                                     |  48%
|
|==================================                                    |  48%
```

```
  |
  |==================================                                    |  49%
  |
  |===================================                                   |  49%
  |
  |===================================                                   |  50%
  |
  |===================================                                   |  51%
  |
  |====================================                                  |  51%
  |
  |====================================                                  |  52%
  |
  |=====================================                                 |  52%
  |
  |=====================================                                 |  53%
  |
  |=====================================                                 |  54%
  |
  |======================================                                |  54%
  |
  |======================================                                |  55%
  |
  |=======================================                               |  55%
  |
  |=======================================                               |  56%
  |
  |========================================                              |  56%
  |
  |========================================                              |  57%
  |
  |========================================                              |  58%
  |
  |=========================================                             |  58%
  |
  |=========================================                             |  59%
  |
  |==========================================                            |  59%
  |
  |==========================================                            |  60%
  |
  |==========================================                            |  61%
  |
  |===========================================                           |  61%
  |
  |===========================================                           |  62%
  |
  |============================================                          |  62%
  |
  |============================================                          |  63%
  |
```

```
|============================================                          | 64%
|
|=============================================                         | 64%
|
|=============================================                         | 65%
|
|==============================================                        | 65%
|
|==============================================                        | 66%
|
|===============================================                       | 66%
|
|===============================================                       | 67%
|
|===============================================                       | 68%
|
|================================================                      | 68%
|
|================================================                      | 69%
|
|=================================================                     | 69%
|
|=================================================                     | 70%
|
|=================================================                     | 71%
|
|==================================================                    | 71%
|
|==================================================                    | 72%
|
|===================================================                   | 72%
|
|===================================================                   | 73%
|
|===================================================                   | 74%
|
|====================================================                  | 74%
|
|====================================================                  | 75%
|
|=====================================================                 | 75%
|
|=====================================================                 | 76%
|
|======================================================                | 76%
|
|======================================================                | 77%
|
|======================================================                | 78%
|
|=======================================================               | 78%
```

```
|
|=======================================================                |  79%
|
|========================================================               |  79%
|
|========================================================               |  80%
|
|========================================================               |  81%
|
|=========================================================              |  81%
|
|=========================================================              |  82%
|
|==========================================================             |  82%
|
|==========================================================             |  83%
|
|==========================================================             |  84%
|
|===========================================================            |  84%
|
|===========================================================            |  85%
|
|============================================================           |  85%
|
|============================================================           |  86%
|
|=============================================================          |  86%
|
|=============================================================          |  87%
|
|=============================================================          |  88%
|
|==============================================================         |  88%
|
|==============================================================         |  89%
|
|===============================================================        |  89%
|
|===============================================================        |  90%
|
|===============================================================        |  91%
|
|================================================================       |  91%
|
|================================================================       |  92%
|
|=================================================================      |  92%
|
|=================================================================      |  93%
|
```

```
  |=================================================================     |  94%
  |
  |==================================================================    |  94%
  |
  |==================================================================    |  95%
  |
  |===================================================================   |  95%
  |
  |===================================================================   |  96%
  |
  |====================================================================  |  96%
  |
  |====================================================================  |  97%
  |
  |====================================================================  |  98%
  |
  |===================================================================== |  98%
  |
  |===================================================================== |  99%
  |
  |======================================================================|  99%
  |
  |======================================================================| 100%
```

```
b2 <- bootnet(network1, boots=1000, type="case", statistics=c("strength", "expectedInfluenc
e", "betweenness", "closeness", "edge"))
```

```
##
  |                                                                      
  |                                                                |   0%
  |                                                                      
  |                                                                |   1%
  |                                                                      
  |=                                                               |   1%
  |                                                                      
  |=                                                               |   2%
  |                                                                      
  |==                                                              |   2%
  |                                                                      
  |==                                                              |   3%
  |                                                                      
  |==                                                              |   4%
  |                                                                      
  |===                                                             |   4%
  |                                                                      
  |===                                                             |   5%
  |                                                                      
  |====                                                            |   5%
  |                                                                      
  |====                                                            |   6%
  |                                                                      
  |=====                                                           |   6%
  |                                                                      
  |=====                                                           |   7%
  |                                                                      
  |=====                                                           |   8%
  |                                                                      
  |======                                                          |   8%
  |                                                                      
  |======                                                          |   9%
  |                                                                      
  |=======                                                         |   9%
  |                                                                      
  |=======                                                         |  10%
  |                                                                      
  |=======                                                         |  11%
  |                                                                      
  |========                                                        |  11%
  |                                                                      
  |========                                                        |  12%
  |                                                                      
  |=========                                                       |  12%
  |                                                                      
  |=========                                                       |  13%
  |                                                                      
  |=========                                                       |  14%
  |                                                                      
  |==========                                                      |  14%
```

```
|
|==========                                                            |  15%
|
|===========                                                           |  15%
|
|===========                                                           |  16%
|
|============                                                          |  16%
|
|============                                                          |  17%
|
|============                                                          |  18%
|
|=============                                                         |  18%
|
|=============                                                         |  19%
|
|==============                                                        |  19%
|
|==============                                                        |  20%
|
|==============                                                        |  21%
|
|===============                                                       |  21%
|
|===============                                                       |  22%
|
|================                                                      |  22%
|
|================                                                      |  23%
|
|================                                                      |  24%
|
|=================                                                     |  24%
|
|=================                                                     |  25%
|
|==================                                                    |  25%
|
|==================                                                    |  26%
|
|===================                                                   |  26%
|
|===================                                                   |  27%
|
|===================                                                   |  28%
|
|====================                                                  |  28%
|
|====================                                                  |  29%
|
```

```
|=====================                                                 |  29%
|
|=====================                                                 |  30%
|
|=====================                                                 |  31%
|
|======================                                                |  31%
|
|======================                                                |  32%
|
|=======================                                               |  32%
|
|=======================                                               |  33%
|
|=======================                                               |  34%
|
|========================                                              |  34%
|
|========================                                              |  35%
|
|=========================                                             |  35%
|
|=========================                                             |  36%
|
|==========================                                            |  36%
|
|==========================                                            |  37%
|
|==========================                                            |  38%
|
|===========================                                           |  38%
|
|===========================                                           |  39%
|
|============================                                          |  39%
|
|============================                                          |  40%
|
|============================                                          |  41%
|
|=============================                                         |  41%
|
|=============================                                         |  42%
|
|==============================                                        |  42%
|
|==============================                                        |  43%
|
|==============================                                        |  44%
|
|===============================                                       |  44%
```

```
|
|===============================                                       |  45%
|
|================================                                      |  45%
|
|================================                                      |  46%
|
|=================================                                     |  46%
|
|=================================                                     |  47%
|
|=================================                                     |  48%
|
|==================================                                    |  48%
|
|==================================                                    |  49%
|
|===================================                                   |  49%
|
|===================================                                   |  50%
|
|===================================                                   |  51%
|
|====================================                                  |  51%
|
|====================================                                  |  52%
|
|=====================================                                 |  52%
|
|=====================================                                 |  53%
|
|=====================================                                 |  54%
|
|======================================                                |  54%
|
|======================================                                |  55%
|
|=======================================                               |  55%
|
|=======================================                               |  56%
|
|========================================                              |  56%
|
|========================================                              |  57%
|
|========================================                              |  58%
|
|=========================================                             |  58%
|
|=========================================                             |  59%
|
```

```
|==========================================                            |  59%
|
|==========================================                            |  60%
|
|==========================================                            |  61%
|
|===========================================                           |  61%
|
|===========================================                           |  62%
|
|============================================                          |  62%
|
|============================================                          |  63%
|
|============================================                          |  64%
|
|=============================================                         |  64%
|
|=============================================                         |  65%
|
|==============================================                        |  65%
|
|==============================================                        |  66%
|
|===============================================                       |  66%
|
|===============================================                       |  67%
|
|===============================================                       |  68%
|
|================================================                      |  68%
|
|================================================                      |  69%
|
|=================================================                     |  69%
|
|=================================================                     |  70%
|
|=================================================                     |  71%
|
|==================================================                    |  71%
|
|==================================================                    |  72%
|
|===================================================                   |  72%
|
|===================================================                   |  73%
|
|===================================================                   |  74%
|
|====================================================                  |  74%
```

```
|
|====================================================                  |  75%
|
|=====================================================                 |  75%
|
|=====================================================                 |  76%
|
|======================================================                |  76%
|
|======================================================                |  77%
|
|======================================================                |  78%
|
|=======================================================               |  78%
|
|=======================================================               |  79%
|
|========================================================              |  79%
|
|========================================================              |  80%
|
|========================================================              |  81%
|
|=========================================================             |  81%
|
|=========================================================             |  82%
|
|==========================================================            |  82%
|
|==========================================================            |  83%
|
|==========================================================            |  84%
|
|===========================================================           |  84%
|
|===========================================================           |  85%
|
|============================================================          |  85%
|
|============================================================          |  86%
|
|=============================================================         |  86%
|
|=============================================================         |  87%
|
|=============================================================         |  88%
|
|==============================================================        |  88%
|
|==============================================================        |  89%
|
```

```
  |===============================================================       |  89%
  |
  |===============================================================       |  90%
  |
  |===============================================================       |  91%
  |
  |================================================================      |  91%
  |
  |================================================================      |  92%
  |
  |=================================================================     |  92%
  |
  |=================================================================     |  93%
  |
  |=================================================================     |  94%
  |
  |==================================================================    |  94%
  |
  |==================================================================    |  95%
  |
  |===================================================================   |  95%
  |
  |===================================================================   |  96%
  |
  |====================================================================  |  96%
  |
  |====================================================================  |  97%
  |
  |====================================================================  |  98%
  |
  |===================================================================== |  98%
  |
  |===================================================================== |  99%
  |
  |======================================================================|  99%
  |
  |======================================================================| 100%
##
  |
  |                                                                      |   0%
  |
  |                                                                      |   1%
  |
  |=                                                                     |   1%
  |
  |=                                                                     |   2%
  |
  |==                                                                    |   2%
  |
  |==                                                                    |   3%
  |
```

```
|==                                                                     |   4%
|
|===                                                                    |   4%
|
|===                                                                    |   5%
|
|====                                                                   |   5%
|
|====                                                                   |   6%
|
|=====                                                                  |   6%
|
|=====                                                                  |   7%
|
|=====                                                                  |   8%
|
|======                                                                 |   8%
|
|======                                                                 |   9%
|
|=======                                                                |   9%
|
|=======                                                                |  10%
|
|=======                                                                |  11%
|
|========                                                               |  11%
|
|========                                                               |  12%
|
|=========                                                              |  12%
|
|=========                                                              |  13%
|
|=========                                                              |  14%
|
|==========                                                             |  14%
|
|==========                                                             |  15%
|
|===========                                                            |  15%
|
|===========                                                            |  16%
|
|============                                                           |  16%
|
|============                                                           |  17%
|
|============                                                           |  18%
|
|=============                                                          |  18%
```

```
|
|=============                                                         |  19%
|
|==============                                                        |  19%
|
|==============                                                        |  20%
|
|==============                                                        |  21%
|
|===============                                                       |  21%
|
|===============                                                       |  22%
|
|================                                                      |  22%
|
|================                                                      |  23%
|
|================                                                      |  24%
|
|=================                                                     |  24%
|
|=================                                                     |  25%
|
|==================                                                    |  25%
|
|==================                                                    |  26%
|
|===================                                                   |  26%
|
|===================                                                   |  27%
|
|===================                                                   |  28%
|
|====================                                                  |  28%
|
|====================                                                  |  29%
|
|=====================                                                 |  29%
|
|=====================                                                 |  30%
|
|=====================                                                 |  31%
|
|======================                                                |  31%
|
|======================                                                |  32%
|
|=======================                                               |  32%
|
|=======================                                               |  33%
|
```

```
|=======================                                               |  34%
|
|========================                                              |  34%
|
|========================                                              |  35%
|
|=========================                                             |  35%
|
|=========================                                             |  36%
|
|==========================                                            |  36%
|
|==========================                                            |  37%
|
|==========================                                            |  38%
|
|===========================                                           |  38%
|
|===========================                                           |  39%
|
|============================                                          |  39%
|
|============================                                          |  40%
|
|============================                                          |  41%
|
|=============================                                         |  41%
|
|=============================                                         |  42%
|
|==============================                                        |  42%
|
|==============================                                        |  43%
|
|==============================                                        |  44%
|
|===============================                                       |  44%
|
|===============================                                       |  45%
|
|================================                                      |  45%
|
|================================                                      |  46%
|
|=================================                                     |  46%
|
|=================================                                     |  47%
|
|=================================                                     |  48%
|
|==================================                                    |  48%
```

```
|
|==================================                                    |  49%
|
|===================================                                   |  49%
|
|===================================                                   |  50%
|
|===================================                                   |  51%
|
|====================================                                  |  51%
|
|====================================                                  |  52%
|
|=====================================                                 |  52%
|
|=====================================                                 |  53%
|
|=====================================                                 |  54%
|
|======================================                                |  54%
|
|======================================                                |  55%
|
|=======================================                               |  55%
|
|=======================================                               |  56%
|
|========================================                              |  56%
|
|========================================                              |  57%
|
|========================================                              |  58%
|
|=========================================                             |  58%
|
|=========================================                             |  59%
|
|==========================================                            |  59%
|
|==========================================                            |  60%
|
|==========================================                            |  61%
|
|===========================================                           |  61%
|
|===========================================                           |  62%
|
|============================================                          |  62%
|
|============================================                          |  63%
|
```

```
|============================================ | 64%
|
|============================================= | 64%
|
|============================================= | 65%
|
|============================================== | 65%
|
|============================================== | 66%
|
|=============================================== | 66%
|
|=============================================== | 67%
|
|=============================================== | 68%
|
|================================================ | 68%
|
|================================================ | 69%
|
|================================================= | 69%
|
|================================================= | 70%
|
|================================================= | 71%
|
|================================================== | 71%
|
|================================================== | 72%
|
|=================================================== | 72%
|
|=================================================== | 73%
|
|=================================================== | 74%
|
|==================================================== | 74%
|
|==================================================== | 75%
|
|===================================================== | 75%
|
|===================================================== | 76%
|
|====================================================== | 76%
|
|====================================================== | 77%
|
|====================================================== | 78%
|
|======================================================= | 78%
```

```
|
|=======================================================               |  79%
|
|========================================================              |  79%
|
|========================================================              |  80%
|
|========================================================              |  81%
|
|=========================================================             |  81%
|
|=========================================================             |  82%
|
|==========================================================            |  82%
|
|==========================================================            |  83%
|
|==========================================================            |  84%
|
|===========================================================           |  84%
|
|===========================================================           |  85%
|
|============================================================          |  85%
|
|============================================================          |  86%
|
|=============================================================         |  86%
|
|=============================================================         |  87%
|
|=============================================================         |  88%
|
|==============================================================        |  88%
|
|==============================================================        |  89%
|
|===============================================================       |  89%
|
|===============================================================       |  90%
|
|===============================================================       |  91%
|
|================================================================      |  91%
|
|================================================================      |  92%
|
|=================================================================     |  92%
|
|=================================================================     |  93%
|
```

```
  |=================================================================     |  94%
  |
  |==================================================================    |  94%
  |
  |==================================================================    |  95%
  |
  |===================================================================   |  95%
  |
  |===================================================================   |  96%
  |
  |====================================================================  |  96%
  |
  |====================================================================  |  97%
  |
  |====================================================================  |  98%
  |
  |===================================================================== |  98%
  |
  |===================================================================== |  99%
  |
  |======================================================================|  99%
  |
  |======================================================================| 100%
```

Centrality Stability Coefficient

```
corStability(b2)
```

```
## === Correlation Stability Analysis ===
##
## Sampling levels tested:
##    nPerson Drop%   n
## 1      141  75.0  98
## 2      185  67.3 117
## 3      229  59.5 107
## 4      273  51.7  95
## 5      317  43.9  82
## 6      361  36.1 107
## 7      405  28.3  92
## 8      449  20.5  97
## 9      493  12.7 103
## 10     537   5.0 102
##
## Maximum drop proportions to retain correlation of 0.7 in at least 95% of the samples:
##
## betweenness: 0
##   - For more accuracy, run bootnet(..., caseMin = 0, caseMax = 0.05)
##
## closeness: 0.205
##   - For more accuracy, run bootnet(..., caseMin = 0.127, caseMax = 0.283)
##
## edge: 0.75 (CS-coefficient is highest level tested)
##   - For more accuracy, run bootnet(..., caseMin = 0.673, caseMax = 1)
##
## expectedInfluence: 0.75 (CS-coefficient is highest level tested)
##   - For more accuracy, run bootnet(..., caseMin = 0.673, caseMax = 1)
##
## strength: 0.673
##   - For more accuracy, run bootnet(..., caseMin = 0.595, caseMax = 0.75)
##
## Accuracy can also be increased by increasing both 'nBoots' and 'caseN'.
```

Plot Centrality Stability Graphs

```
plot(b2)
```

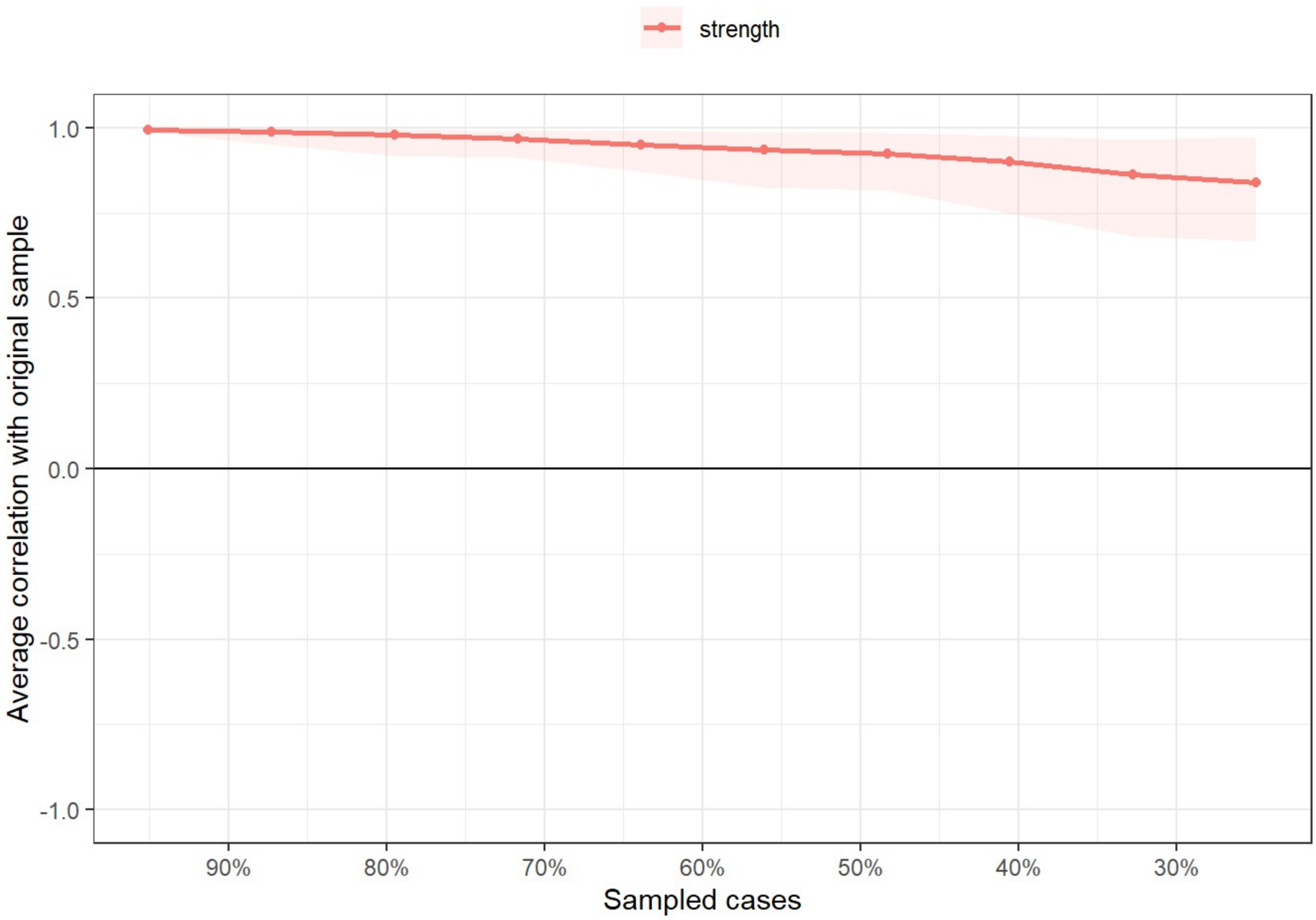


```
dev.off()
```

```
## null device
##           1
```

```
plot(b2, "ExpectedInfluence")
dev.off()
```

```
## null device
##           1
```

```
plot(b2, "betweenness")
dev.off()
```

```
## null device
##           1
```

```
plot(b2, "closeness")
dev.off()
```

```
## null device
##           1
```

Strength Centrality Diff Test

```
plot(b1, "strength", order="sample", labels=TRUE)
```

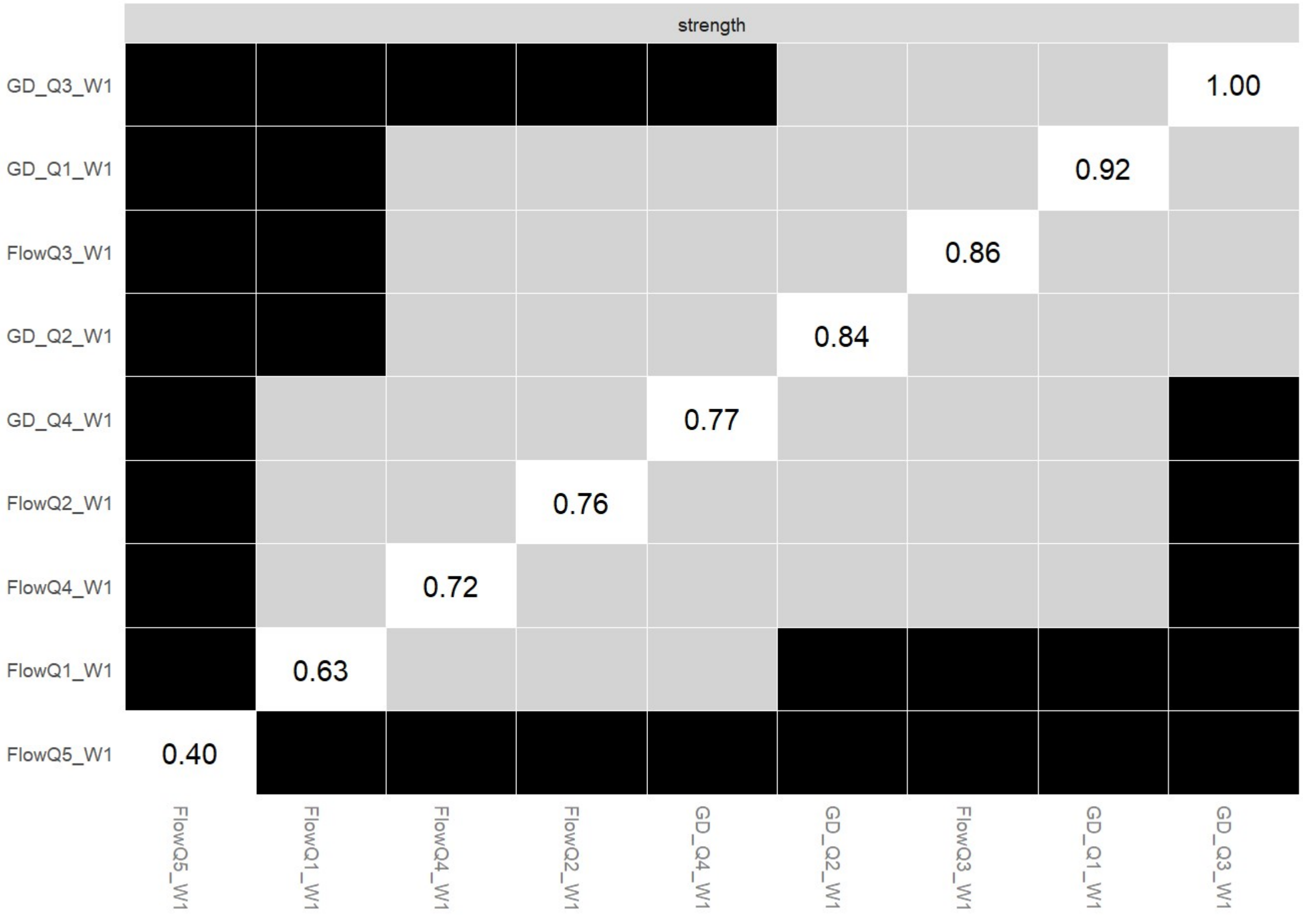


```
dev.off()
```

```
## null device
##           1
```

Expected Inflience Centrality Diff test

```
plot(b1, "expectedInfluence", order="sample", labels=TRUE)
```

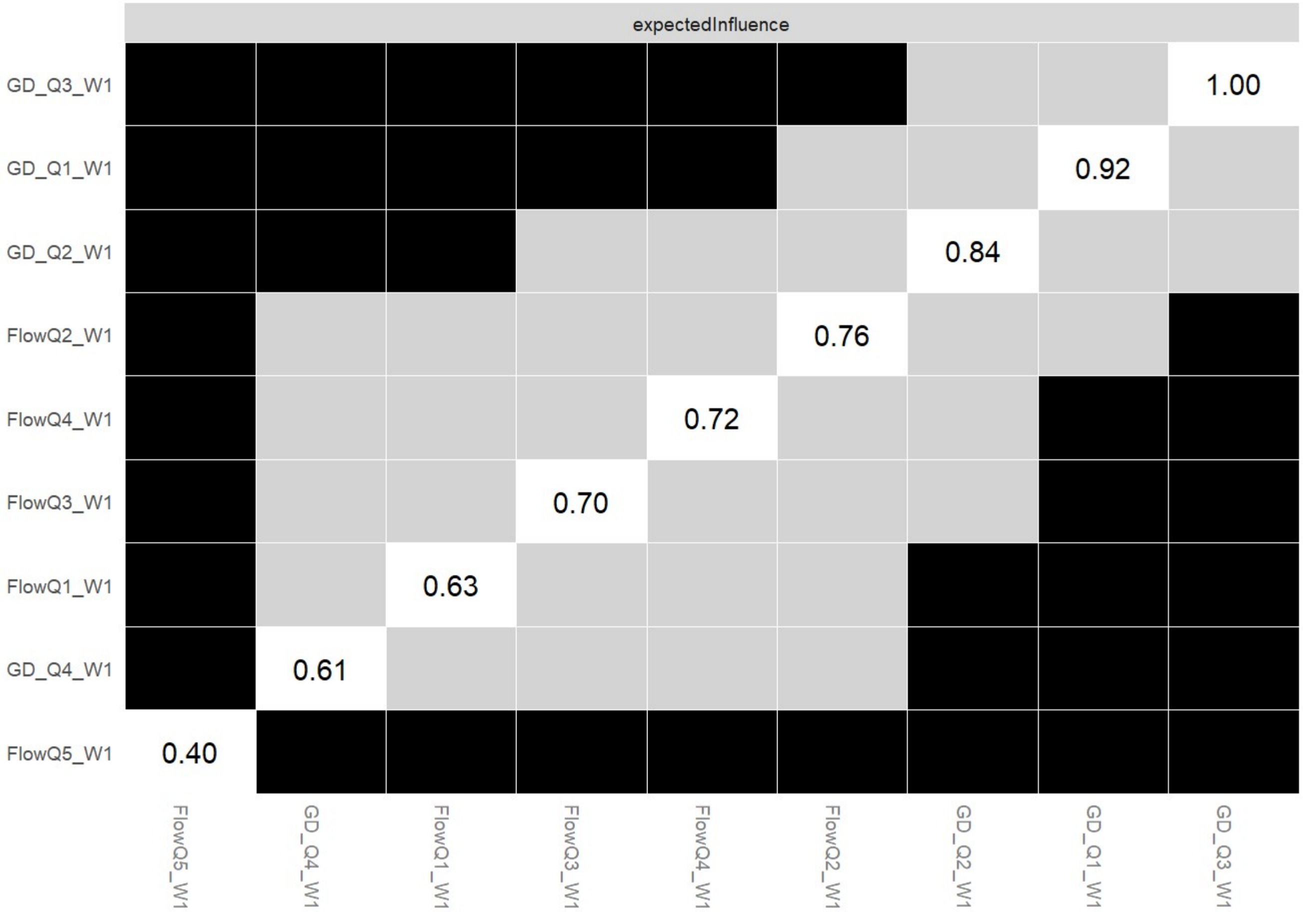


```
dev.off()
```

```
## null device
##           1
```

Edge Stability Graph

```
plot(b1, labels = FALSE, order = "sample")
```

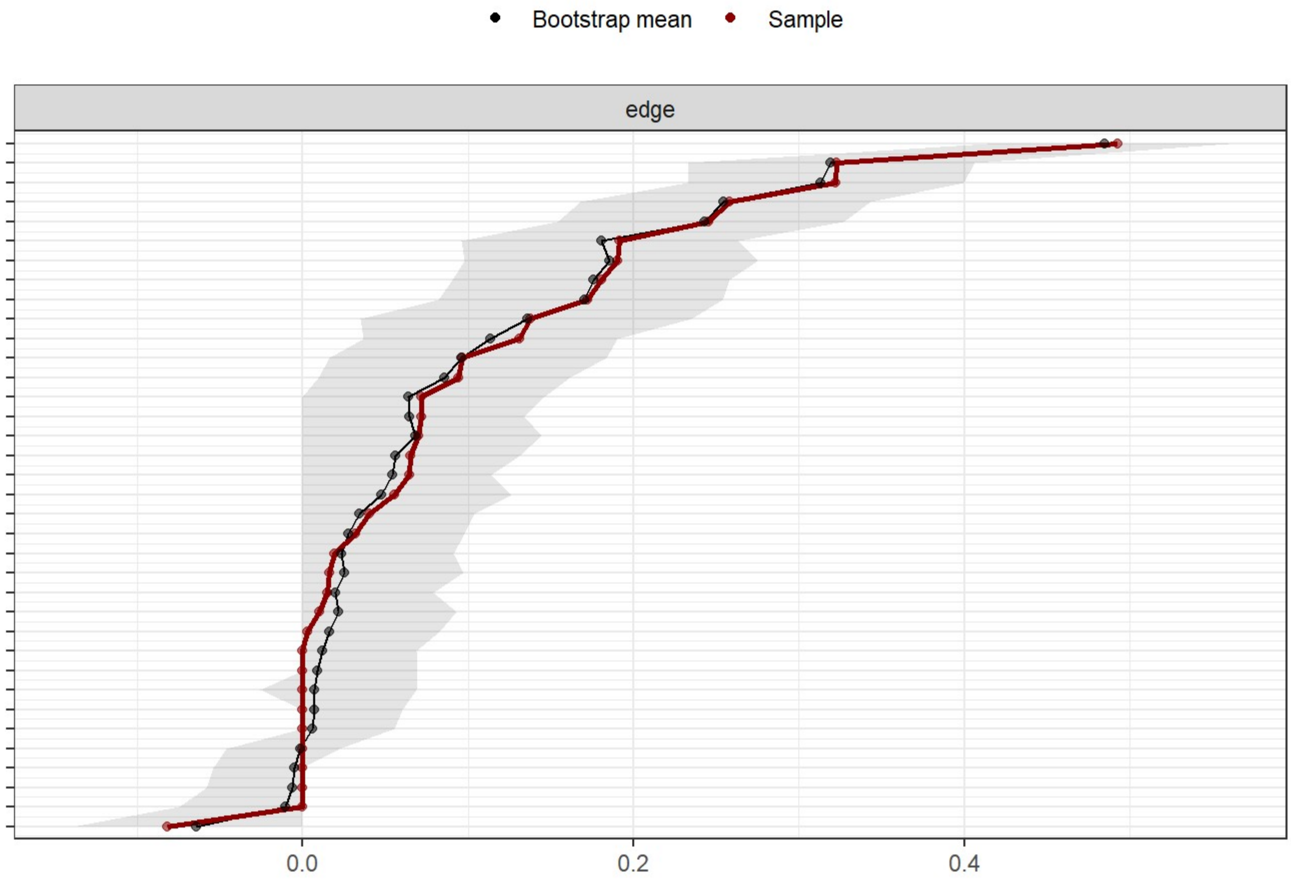


```
dev.off()
```

```
## null device
##           1
```

Edge Weights Stability

```
plot(b1, "edge", plot="difference", onlyNonZero=TRUE, order = "sample")
```

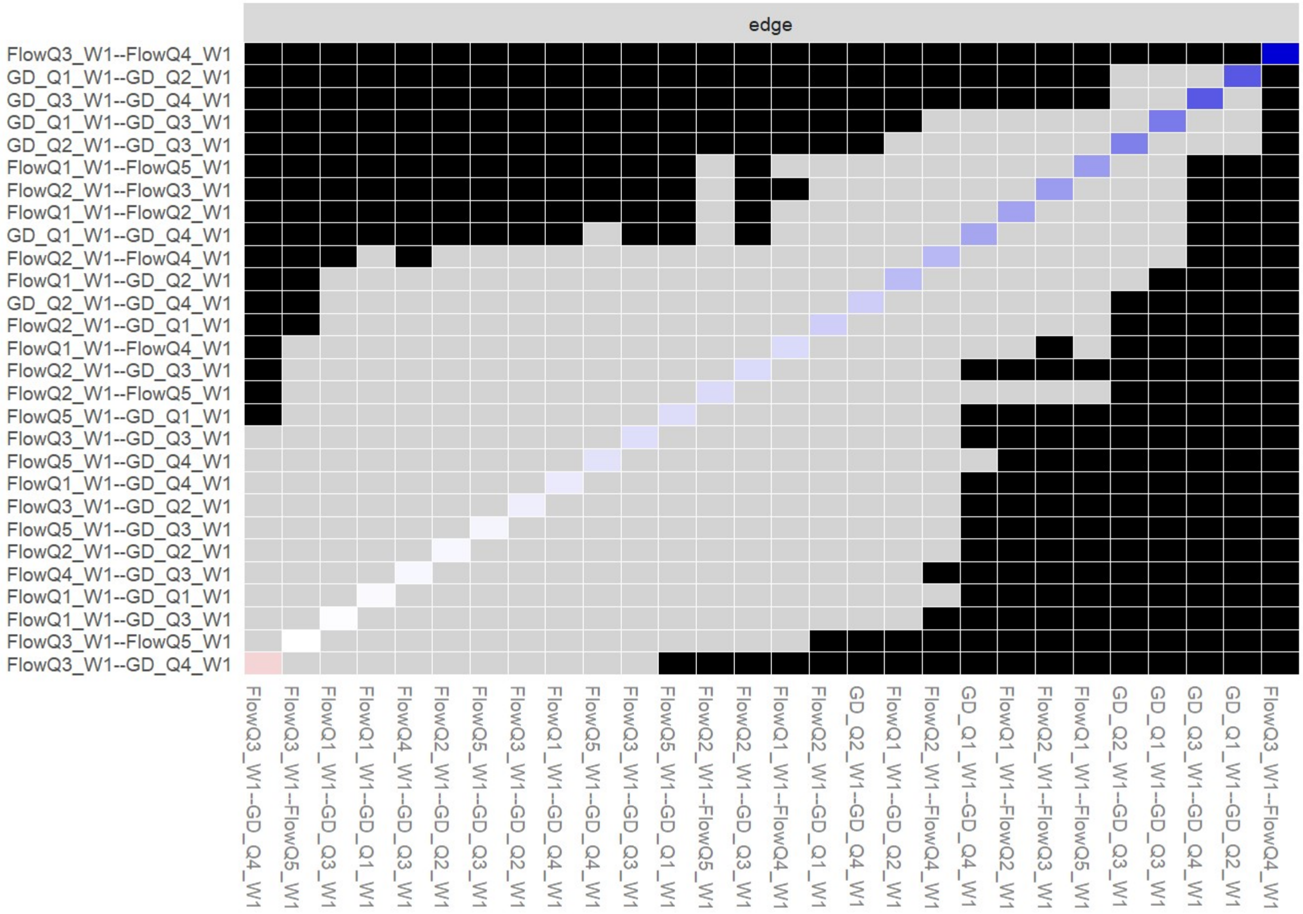


```
dev.off()
```

```
## null device
##           1
```

Strength/Expected Influence Centrality Plots

```
c1 <- centralityPlot(network1, include = c("ExpectedInfluence"), orderBy ="default")
```

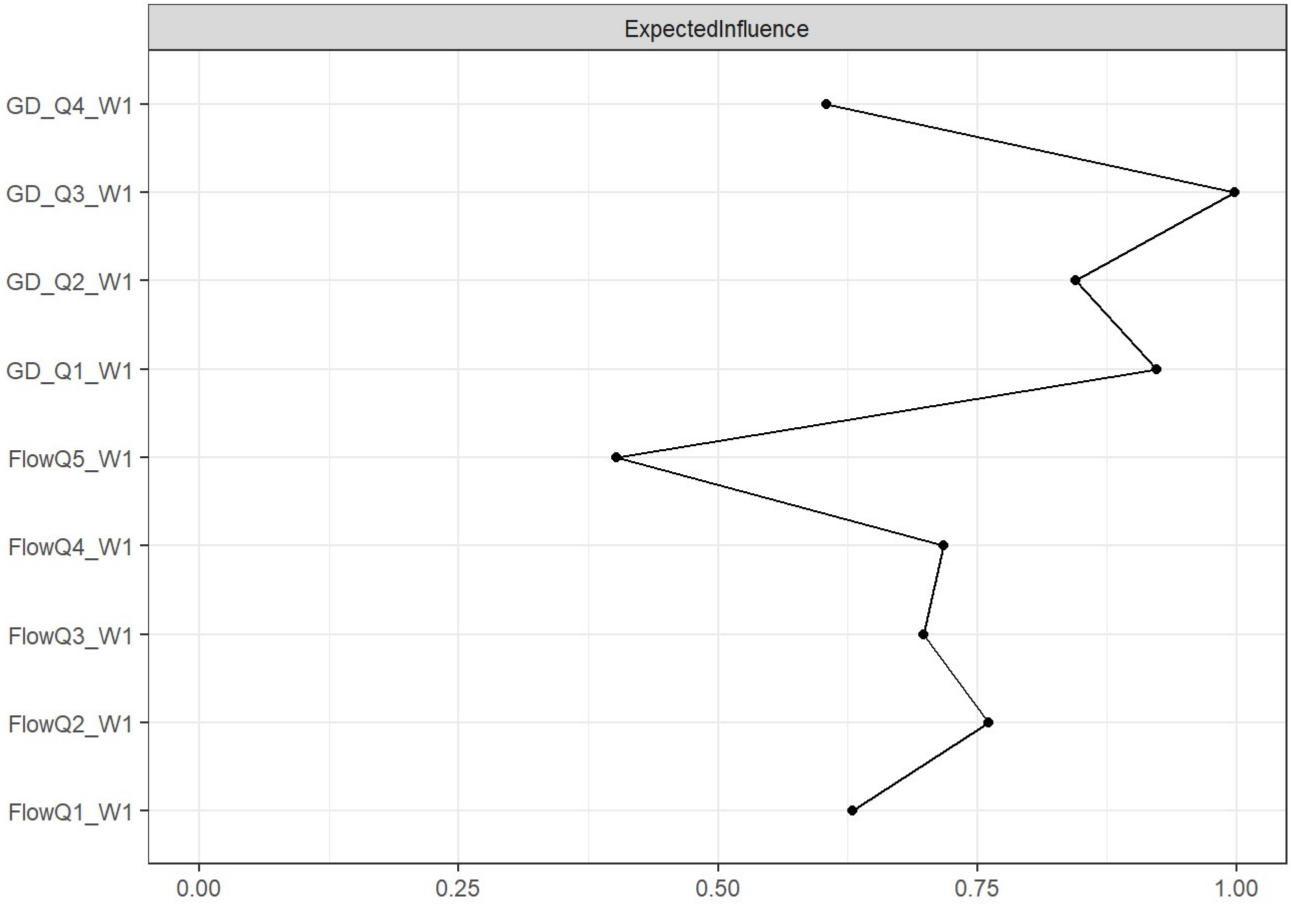


```
dev.off()
```

```
## null device
##           1
```

```
c2 <- centralityPlot(network1, include = c("Betweenness", "Closeness"), orderBy ="default")
dev.off()
```

```
## null device
##           1
```

Edge Strength/Correlations

```
edges1<-getWmat(network1)
write.csv(edges1, "edges1.csv")
```

Inspect Centrality Indices

```
Centrality1 <- centralityTable(network1)
write.csv(Centrality1, "Centrality1.csv")
view(Centrality1)
```

Network Plot With Groups

```
plot1 <- plot(network1, layout="spring", vsize=6, border.color="black", groups=groups1, color
=c('lightblue', 'orange'),labels=names)
```

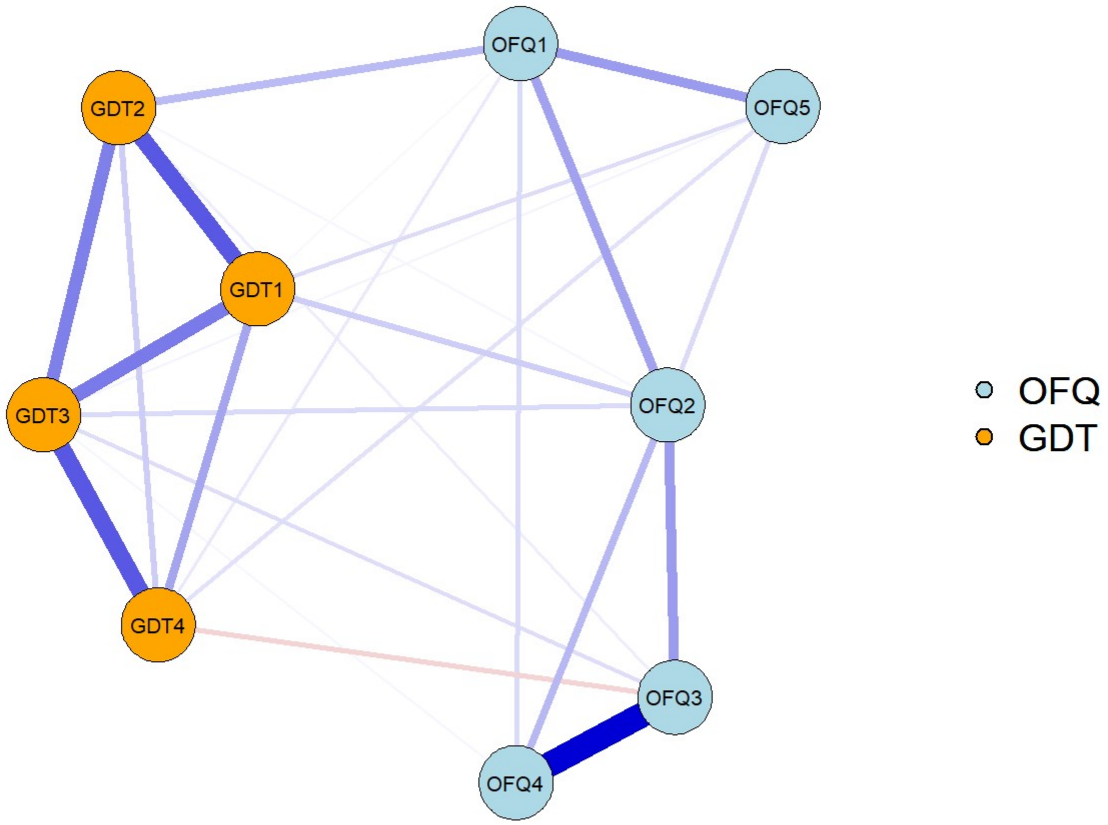


```
dev.off()
```

```
## null device
##           1
```

```
plot1 <- plot(network1, layout="spring", vsize=6, border.color="black", groups=groups1, color
=c('lightblue', 'orange'))
dev.off()
```

```
## null device
##           1
```

```
plot1 <- plot(network1, layout="spring", vsize=6, border.color="black", groups=groups1, color
=c('lightblue', 'orange'),labels=names1)
dev.off()
```

```
## null device
##           1
```

Estimate Bridge Values

```
bridge1<-bridge(plot1, communities=c('1', '1', '1', '1', '1', '2', '2', '2', '2'), useCommuni
ties = "all", directed = NULL, nodes = NULL)
view(bridge1)
```

Bridge Strength Plot

```
pdf("bridgecentrality1.pdf", width = 5)
plot(bridge1, include = "Bridge Strength")
dev.off()
```

```
## png
##   2
```

Flow PCA

[redacted]

[redacted]

```
library(haven) #For importing data
library(psych)  # For PCA with rotation
library(ggplot2)  # For visualization

##
## Attaching package: 'ggplot2'

## The following objects are masked from 'package:psych':
##
##     %+%, alpha

library(GPArotation) #For rotation

##
## Attaching package: 'GPArotation'

## The following objects are masked from 'package:psych':
##
##     equamax, varimin

# Select only the relevant Flow variables for PCA
flow_pca_data <- flow_data[, c("Flow1", "Flow2", "Flow3", "Flow4", "Flow5")]

# Standardize the data (PCA works best with standardized variables)
flow_pca_data <- scale(flow_pca_data)

# Conduct PCA with Direct Oblimin rotation
pca_result <- principal(flow_pca_data, nfactors = 5, rotate = "oblimin",
scores = TRUE)

# Print PCA results
print(pca_result)

## Principal Components Analysis
## Call: principal(r = flow_pca_data, nfactors = 5, rotate = "oblimin",
##     scores = TRUE)
## Standardized loadings (pattern matrix) based upon correlation matrix
##       TC3 TC2 TC1 TC4 TC5 h2       u2 com
## Flow1   1   0   0   0   0  1 -4.4e-16   1
## Flow2   0   0   0   1   0  1 -6.7e-16   1
## Flow3   0   0   0   0   1  1  2.2e-16   1
## Flow4   0   0   1   0   0  1 -6.7e-16   1
## Flow5   0   1   0   0   0  1  1.1e-16   1
##
```

```
##                         TC3 TC2 TC1 TC4 TC5
## SS loadings             1.0 1.0 1.0 1.0 1.0
## Proportion Var          0.2 0.2 0.2 0.2 0.2
## Cumulative Var          0.2 0.4 0.6 0.8 1.0
## Proportion Explained  0.2 0.2 0.2 0.2 0.2
## Cumulative Proportion 0.2 0.4 0.6 0.8 1.0
##
##  With component correlations of
##      TC3  TC2  TC1  TC4  TC5
## TC3 1.00 0.28 0.21 0.32 0.16
## TC2 0.28 1.00 0.09 0.20 0.04
## TC1 0.21 0.09 1.00 0.35 0.58
## TC4 0.32 0.20 0.35 1.00 0.38
## TC5 0.16 0.04 0.58 0.38 1.00
##
## Mean item complexity =  1
## Test of the hypothesis that 5 components are sufficient.
##
## The root mean square of the residuals (RMSR) is  0
##  with the empirical chi square  0  with prob <  NA
##
## Fit based upon off diagonal values = 1
```

```
# Create a table with factor loadings
loadings_table <- data.frame(
  Item = rownames(pca_result$loadings),
  pca_result$loadings[]
)

# Create a table for the proportion of variance explained by each factor
variance_table <- data.frame(
  Component = seq_along(pca_result$values),
  Variance_Explained = pca_result$values / sum(pca_result$values) * 100
)

# Compute correlation matrix for extracted components
component_correlation <- pca_result$Structure


# Display results
print("Factor Loadings Table:")
```

```
## [1] "Factor Loadings Table:"
```

```
print(loadings_table)
```

```
##         Item           TC3           TC2           TC1           TC4
## Flow1 Flow1  1.000002e+00 -3.812015e-07 -1.842206e-06 -1.705960e-06
## Flow2 Flow2  7.008377e-08  5.452963e-08 -9.956264e-07  1.000000e+00
## Flow3 Flow3  4.956477e-06  4.611969e-06  1.742385e-06  3.842376e-06
```

```
## Flow4 Flow4 -8.566008e-08 -9.522741e-08  1.000000e+00 -8.048189e-08
## Flow5 Flow5 -1.424725e-06  1.000001e+00 -1.857136e-06 -2.034970e-06
##                 TC5
## Flow1 -2.999225e-06
## Flow2  9.221628e-07
## Flow3  9.999966e-01
## Flow4 -2.306433e-08
## Flow5 -3.247300e-06
```

```
print("Proportion of Variance Explained by Each Factor:")
```

```
## [1] "Proportion of Variance Explained by Each Factor:"
```

```
print(variance_table)
```

```
##   Component Variance_Explained
## 1         1          42.032008
## 2         2          23.170810
## 3         3          14.169267
## 4         4          12.458472
## 5         5           8.169443
```

```
print("Correlation Matrix Between Extracted Components:")
```

```
## [1] "Correlation Matrix Between Extracted Components:"
```

```
print(component_correlation)
```

```
##             TC3        TC2        TC1       TC4       TC5
## Flow1 1.0000000 0.28408995 0.20738901 0.3190658 0.1551905
## Flow2 0.3190689 0.19954769 0.35352656 1.0000000 0.3762733
## Flow3 0.1552023 0.04042299 0.58423813 0.3762787 1.0000000
## Flow4 0.2073928 0.09442226 1.00000000 0.3535269 0.5842355
## Flow5 0.2840879 1.00000000 0.09441772 0.1995435 0.0404109
```

```
# Scree plot to visualize explained variance
screeplot_result <- data.frame(
  Component = seq_along(pca_result$values),
  Variance = pca_result$values
)

ggplot(screeplot_result, aes(x = Component, y = Variance)) +
  geom_line() +
  geom_point() +
  labs(title = "Scree Plot of PCA", x = "Principal Components", y =
"Eigenvalues") +
  theme_minimal()
```

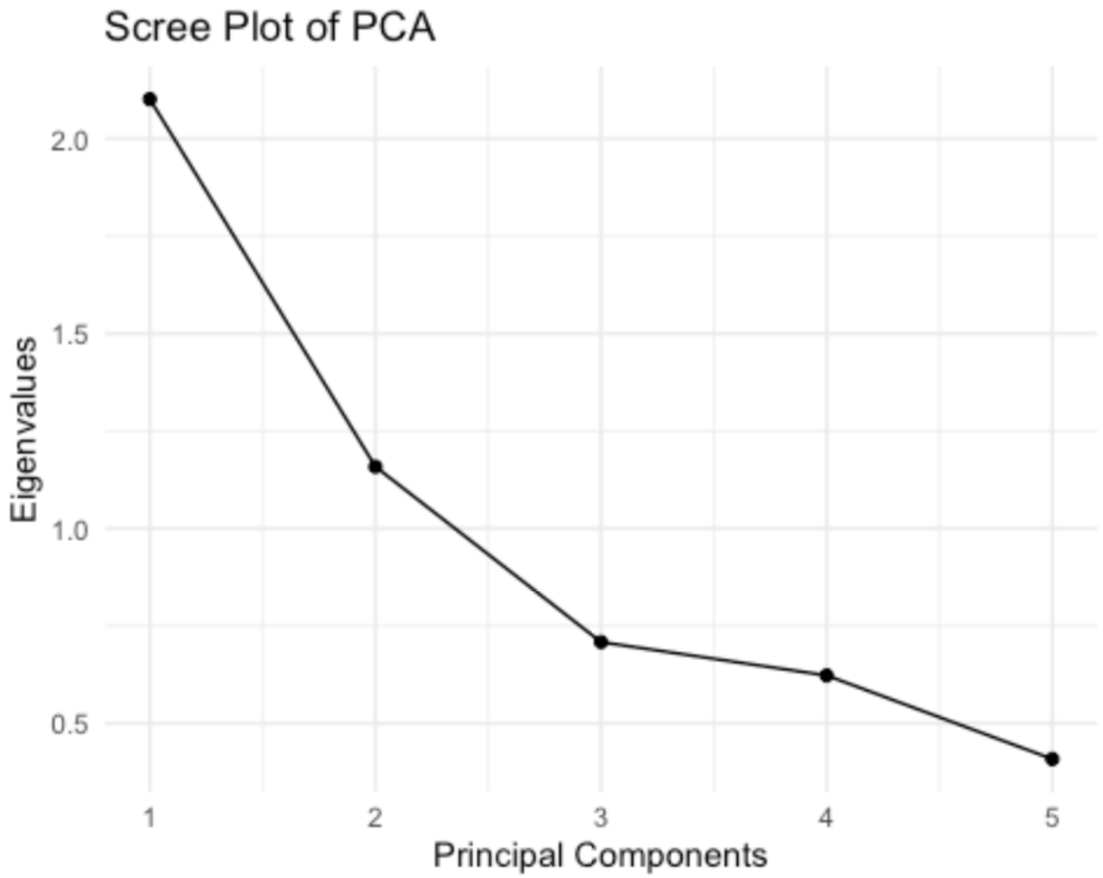


```
# Conduct Parallel Analysis to determine the optimal number of factors
parallel_result <- fa.parallel(flow_pca_data, fa = "pc", n.iter = 100, main =
"Parallel Analysis Scree Plot")
```

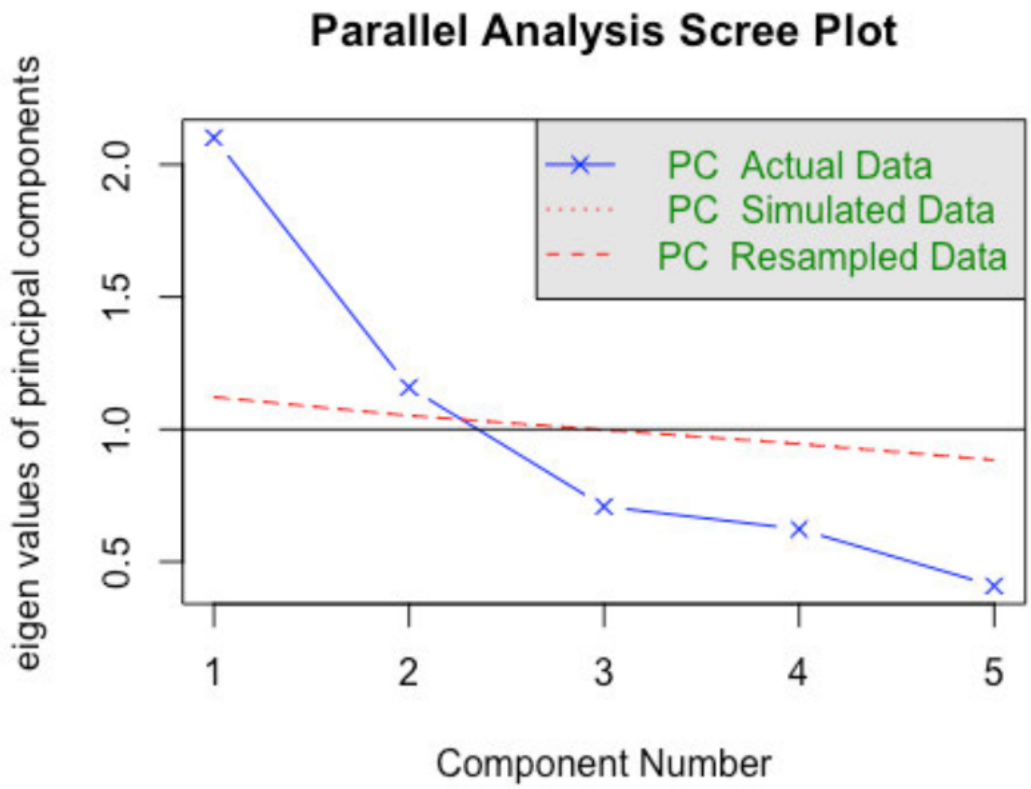


```
## Parallel analysis suggests that the number of factors =  NA  and the
number of components =  2

# Identify the suggested number of components
optimal_factors <- sum(parallel_result$fa.values > parallel_result$fa.sim)

print(paste("Optimal number of components based on Parallel Analysis:",
optimal_factors))

## [1] "Optimal number of components based on Parallel Analysis: NA"

# Conduct PCA with Direct Oblimin rotation using the optimal number of
factors
ppca_result <- principal(flow_pca_data, nfactors=2, rotate = "oblimin",
scores = TRUE)

# Print PCA results
print(ppca_result)

## Principal Components Analysis
## Call: principal(r = flow_pca_data, nfactors = 2, rotate = "oblimin",
##     scores = TRUE)
## Standardized loadings (pattern matrix) based upon correlation matrix
##         TC1   TC2   h2   u2 com
```

```
## Flow1  0.13  0.73 0.59 0.41 1.1
## Flow2  0.55  0.40 0.53 0.47 1.8
## Flow3  0.88 -0.08 0.76 0.24 1.0
## Flow4  0.85  0.01 0.72 0.28 1.0
## Flow5 -0.13  0.82 0.66 0.34 1.1
##
##                        TC1  TC2
## SS loadings           1.85 1.41
## Proportion Var        0.37 0.28
## Cumulative Var        0.37 0.65
## Proportion Explained  0.57 0.43
## Cumulative Proportion 0.57 1.00
##
##  With component correlations of
##      TC1  TC2
## TC1 1.00 0.17
## TC2 0.17 1.00
##
## Mean item complexity =  1.2
## Test of the hypothesis that 2 components are sufficient.
##
## The root mean square of the residuals (RMSR) is  0.14
##  with the empirical chi square  235.76  with prob <  3.3e-53
##
## Fit based upon off diagonal values = 0.77
```

```
# Create a table with factor loadings
loadings_table <- data.frame(
  Item = rownames(ppca_result$loadings),
  pca_result$loadings[]
)

# Create a table for the proportion of variance explained by each factor
variance_table <- data.frame(
  Component = seq_along(ppca_result$values),
  Variance_Explained = ppca_result$values / sum(ppca_result$values) * 100
)

# Compute correlation matrix for extracted components
component_correlation <- ppca_result$Structure

# Display results
print("Factor Loadings Table:")
```

```
## [1] "Factor Loadings Table:"
```

```
print(loadings_table)
```

```
##        Item           TC3           TC2           TC1           TC4
## Flow1 Flow1  1.000002e+00 -3.812015e-07 -1.842206e-06 -1.705960e-06
```

```
## Flow2 Flow2  7.008377e-08  5.452963e-08 -9.956264e-07  1.000000e+00
## Flow3 Flow3  4.956477e-06  4.611969e-06  1.742385e-06  3.842376e-06
## Flow4 Flow4 -8.566008e-08 -9.522741e-08  1.000000e+00 -8.048189e-08
## Flow5 Flow5 -1.424725e-06  1.000001e+00 -1.857136e-06 -2.034970e-06
##                 TC5
## Flow1 -2.999225e-06
## Flow2  9.221628e-07
## Flow3  9.999966e-01
## Flow4 -2.306433e-08
## Flow5 -3.247300e-06

print("Proportion of Variance Explained by Each Factor:")

## [1] "Proportion of Variance Explained by Each Factor:"

print(variance_table)

##   Component Variance_Explained
## 1         1          42.032008
## 2         2          23.170810
## 3         3          14.169267
## 4         4          12.458472
## 5         5           8.169443

print("Correlation Matrix Between Extracted Components:")

## [1] "Correlation Matrix Between Extracted Components:"

print(component_correlation)

##               TC1        TC2
## Flow1 0.260328684 0.75719126
## Flow2 0.614602699 0.49425332
## Flow3 0.868591048 0.07101841
## Flow4 0.846993789 0.15299175
## Flow5 0.009148989 0.80063033

# Scree plot to visualize explained variance
screeplot_result <- data.frame(
  Component = seq_along(ppca_result$values),
  Variance = ppca_result$values
)

ggplot(screeplot_result, aes(x = Component, y = Variance)) +
  geom_line() +
  geom_point() +
  labs(title = "Scree Plot of PCA", x = "Principal Components", y =
"Eigenvalues") +
  theme_minimal()
```

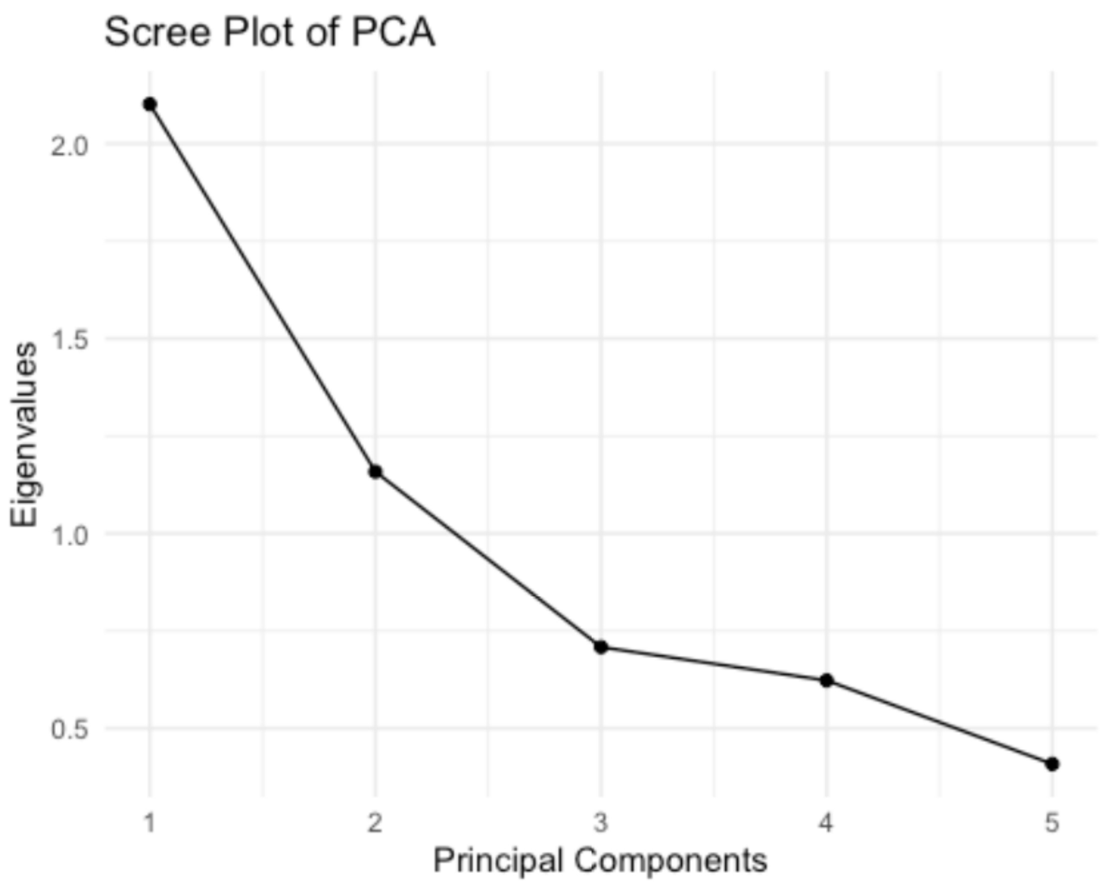


```r
# Conduct Parallel Analysis to determine the optimal number of factors
parallel_result <- fa.parallel(flow_pca_data, fa = "pc", n.iter = 100, main =
"Parallel Analysis Scree Plot")
```

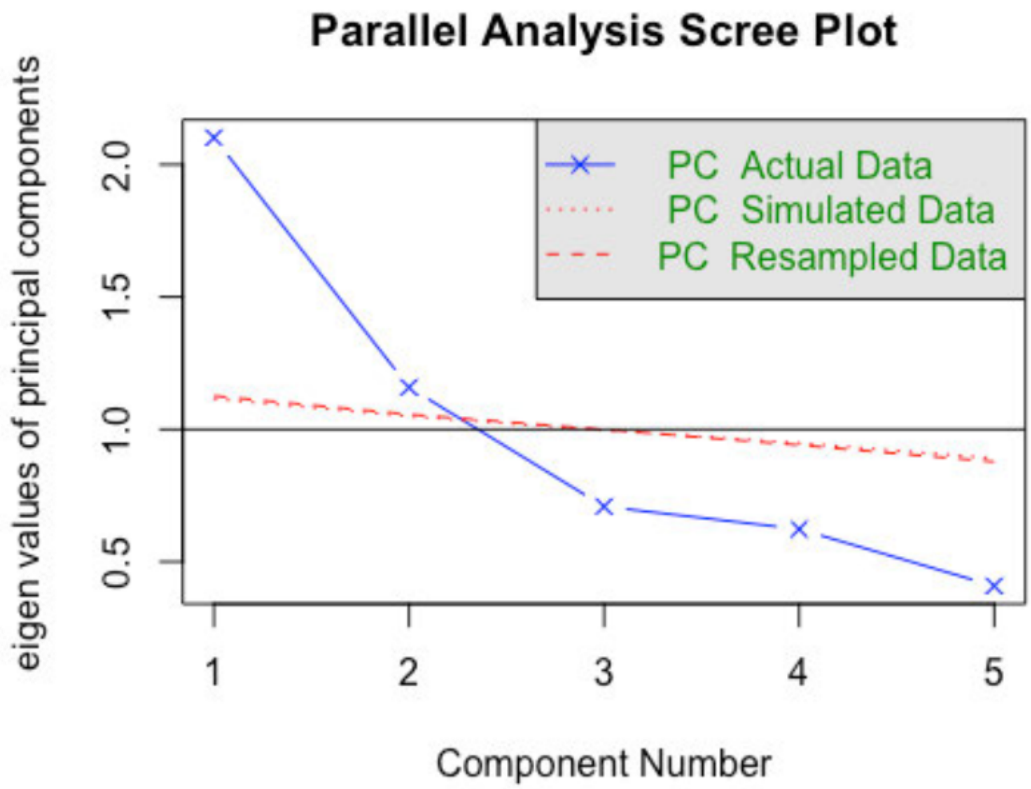


```
## Parallel analysis suggests that the number of factors =  NA  and the
number of components =  2

# Identify the suggested number of components
optimal_factors <- sum(parallel_result$fa.values > parallel_result$fa.sim)

print(paste("Optimal number of components based on Parallel Analysis:",
optimal_factors))

## [1] "Optimal number of components based on Parallel Analysis: NA"

# Conduct PCA with Direct Oblimin rotation using the optimal number of
factors
ppca1_result <- principal(flow_pca_data, nfactors = 1, rotate = "oblimin",
scores = TRUE)

# Print PCA results
print(ppca1_result)

## Principal Components Analysis
## Call: principal(r = flow_pca_data, nfactors = 1, rotate = "oblimin",
##     scores = TRUE)
## Standardized loadings (pattern matrix) based upon correlation matrix
##        PC1   h2   u2 com
```

```
## Flow1 0.55 0.31 0.69   1
## Flow2 0.73 0.53 0.47   1
## Flow3 0.74 0.55 0.45   1
## Flow4 0.76 0.58 0.42   1
## Flow5 0.37 0.13 0.87   1
##
##                  PC1
## SS loadings    2.10
## Proportion Var 0.42
##
## Mean item complexity =  1
## Test of the hypothesis that 1 component is sufficient.
##
## The root mean square of the residuals (RMSR) is  0.17
##  with the empirical chi square  321.55  with prob <  2.3e-67
##
## Fit based upon off diagonal values = 0.69
```

```
# Create a table with factor loadings
loadings_table <- data.frame(
  Item = rownames(ppca1_result$loadings),
  pca_result$loadings[]
)

# Create a table for the proportion of variance explained by each factor
variance_table <- data.frame(
  Component = seq_along(ppca1_result$values),
  Variance_Explained = ppca1_result$values / sum(ppca1_result$values) * 100
)

# Compute correlation matrix for extracted components
component_correlation <- ppca1_result$Structure

# Display results
print("Factor Loadings Table:")
```

```
## [1] "Factor Loadings Table:"
```

```
print(loadings_table)
```

```
##         Item           TC3           TC2           TC1           TC4
## Flow1 Flow1  1.000002e+00 -3.812015e-07 -1.842206e-06 -1.705960e-06
## Flow2 Flow2  7.008377e-08  5.452963e-08 -9.956264e-07  1.000000e+00
## Flow3 Flow3  4.956477e-06  4.611969e-06  1.742385e-06  3.842376e-06
## Flow4 Flow4 -8.566008e-08 -9.522741e-08  1.000000e+00 -8.048189e-08
## Flow5 Flow5 -1.424725e-06  1.000001e+00 -1.857136e-06 -2.034970e-06
##                 TC5
## Flow1 -2.999225e-06
## Flow2  9.221628e-07
## Flow3  9.999966e-01
```

```
## Flow4 -2.306433e-08
## Flow5 -3.247300e-06

print("Proportion of Variance Explained by Each Factor:")

## [1] "Proportion of Variance Explained by Each Factor:"

print(variance_table)

##   Component Variance_Explained
## 1         1          42.032008
## 2         2          23.170810
## 3         3          14.169267
## 4         4          12.458472
## 5         5           8.169443

print("Correlation Matrix Between Extracted Components:")

## [1] "Correlation Matrix Between Extracted Components:"

print(component_correlation)

##
## Loadings:
##       PC1
## Flow1 0.553
## Flow2 0.725
## Flow3 0.744
## Flow4 0.763
## Flow5 0.367
##
##                  PC1
## SS loadings    2.102
## Proportion Var 0.420

# Scree plot to visualize explained variance
screeplot_result <- data.frame(
  Component = seq_along(ppca1_result$values),
  Variance = ppca1_result$values
)

ggplot(screeplot_result, aes(x = Component, y = Variance)) +
  geom_line() +
  geom_point() +
  labs(title = "Scree Plot of PCA", x = "Principal Components", y =
"Eigenvalues") +
  theme_minimal()
```

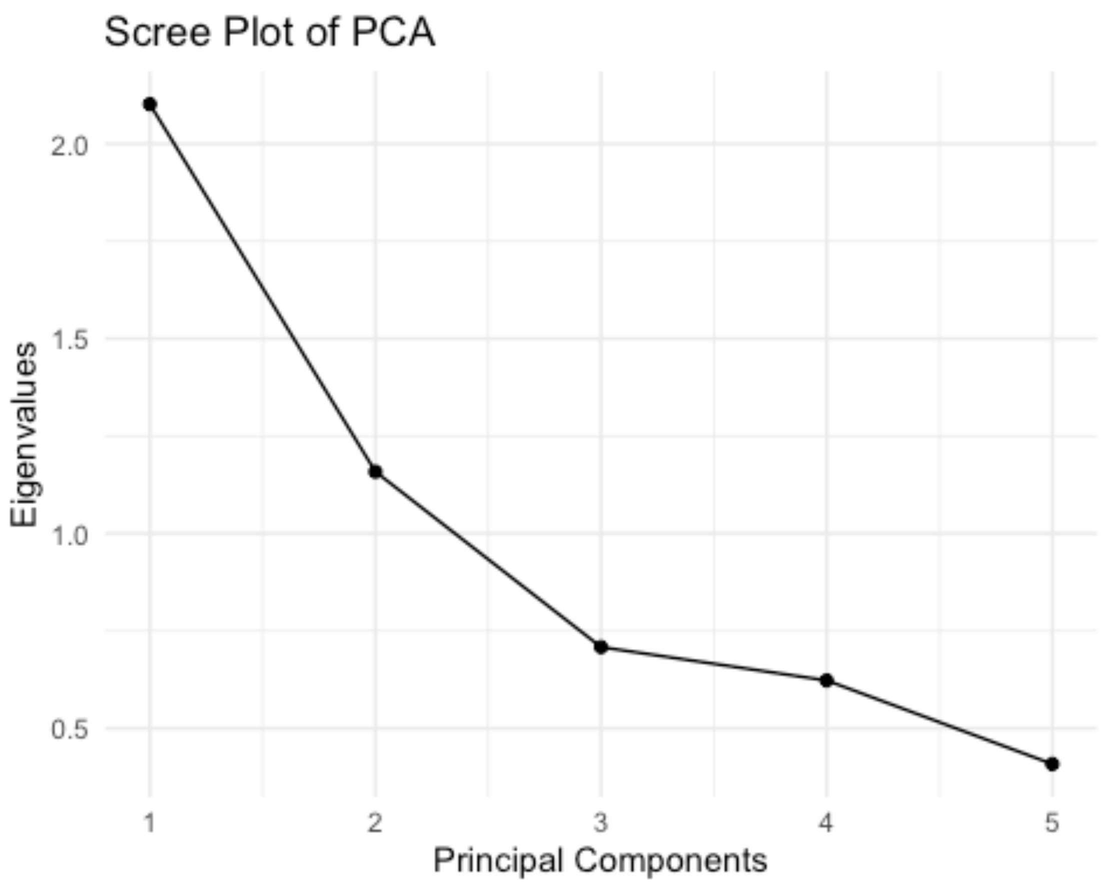
Scree Plot of PCA
Eigenvalues
2.0
1.5
1.0
0.5
1
2
3
4
5
Principal Components
